# Etiology and Risk Factors for Mortality in an Adult Community-acquired Pneumonia Cohort in Malawi

**DOI:** 10.1164/rccm.201807-1333OC

**Published:** 2019-08-01

**Authors:** Stephen J. Aston, Antonia Ho, Hannah Jary, Jacqueline Huwa, Tamara Mitchell, Sarah Ibitoye, Simon Greenwood, Elizabeth Joekes, Arthur Daire, Jane Mallewa, Dean Everett, Mulinda Nyirenda, Brian Faragher, Henry C. Mwandumba, Robert S. Heyderman, Stephen B. Gordon

**Affiliations:** ^1^ Institute of Infection and Global Health, University of Liverpool, Liverpool, United Kingdom; ^2^ Malawi-Liverpool-Wellcome Trust Clinical Research Programme, College of Medicine, Blantyre, Malawi; ^3^ Liverpool School of Tropical of Medicine, Liverpool, United Kingdom; ^4^ Medical Research Council–University of Glasgow Centre for Virus Research, Glasgow, United Kingdom; ^5^ Department of Radiology, Royal Liverpool University Hospital NHS Trust, Liverpool, United Kingdom; ^6^ Queen Elizabeth Central Hospital, Blantyre, Malawi; ^7^ College of Medicine, University of Malawi, Blantyre, Malawi; ^8^ Queens Medical Research Institute, University of Edinburgh, Edinburgh, United Kingdom; and; ^9^ Division of Infection and Immunity, University College London, London, United Kingdom

**Keywords:** community-acquired pneumonia, HIV, Africa south of the Sahara, *Streptococcus pneumoniae*, pulmonary tuberculosis

## Abstract

**Rationale:** In the context of rapid antiretroviral therapy rollout and an increasing burden of noncommunicable diseases, there are few contemporary data describing the etiology and outcome of community-acquired pneumonia (CAP) in sub-Saharan Africa.

**Objectives:** To describe the current etiology of CAP in Malawi and identify risk factors for mortality.

**Methods:** We conducted a prospective observational study of adults hospitalized with CAP to a teaching hospital in Blantyre, Malawi. Etiology was defined by blood culture, *Streptococcus pneumoniae* urinary antigen detection, sputum mycobacterial culture and Xpert MTB/RIF, and nasopharyngeal aspirate multiplex PCR.

**Measurements and Main Results:** In 459 patients (285 [62.1%] males; median age, 34.7 [interquartile range, 29.4–41.9] yr), 30-day mortality was 14.6% (64/439) and associated with male sex (adjusted odds ratio, 2.60 [95% confidence interval, 1.17–5.78]), symptom duration greater than 7 days (2.78 [1.40–5.54]), tachycardia (2.99 [1.48–6.06]), hypoxemia (4.40 [2.03–9.51]), and inability to stand (3.59 [1.72–7.50]). HIV was common (355/453; 78.4%), frequently newly diagnosed (124/355; 34.9%), but not associated with mortality. *S. pneumoniae* (98/458; 21.4%) and *Mycobacterium tuberculosis* (75/326; 23.0%) were the most frequently identified pathogens. Viral infection occurred in 32.6% (148/454) with influenza (40/454; 8.8%) most common. Bacterial–viral coinfection occurred in 9.1% (28/307). Detection of *M. tuberculosis* was associated with mortality (adjusted odds ratio, 2.44 [1.19–5.01]).

**Conclusions:** In the antiretroviral therapy era, CAP in Malawi remains predominantly HIV associated, with a large proportion attributable to potentially vaccine-preventable pathogens. Strategies to increase early detection and treatment of tuberculosis and improve supportive care, in particular the correction of hypoxemia, should be evaluated in clinical trials to address CAP-associated mortality.

**At a Glance Commentary**

**Scientific Knowledge on the Subject**

In the antiretroviral therapy era, community-acquired pneumonia (CAP) remains the commonest cause of adult hospitalization and a major cause of mortality in many sub-Saharan Africa HIV-affected countries. With the exception of cohorts from South Africa, contemporary studies describing CAP etiology and outcome from the region are either small, retrospective, or only use limited investigations to define etiology. Current CAP management protocols applied in sub-Saharan Africa are therefore inadequately informed by contemporary data.

**What This Study Adds to the Field**

In a large prospective cohort of hospitalized adults in Malawi we show that the major burden of hospitalized acute CAP remains in young, HIV-infected patients and that *Streptococcus pneumoniae*, tuberculosis, and influenza predominate as the major causes. CAP mortality is high compared with populations from well-resourced settings with similar age profiles and is associated with potentially modifiable risk factors including hypoxemia. Death from CAP is poorly predicted by commonly used CAP severity-assessment tools, such as CURB65.

Globally, pneumonia is the commonest infectious cause of death and the second leading cause of overall life years lost (1). In sub-Saharan Africa alone, lower respiratory tract infections account for 390,000 deaths in older children and adults each year (2). Although the etiology and outcome of community-acquired pneumonia (CAP) has been thoroughly investigated in large cohorts of adults in well-resourced settings in Europe and North America (3, 4) and more recently in young children (<5 yr) from sub-Saharan Africa (5), contemporary data on acute CAP in adults from sub-Saharan Africa are limited. With the exception of cohorts from South Africa (6, 7), recent studies are small (8, 9), retrospective (10), or only use a restricted panel of investigations to define etiology (11).

In Malawi, a very-low-income country in Southern Africa, pneumonia is the commonest cause of adult hospitalization (12). There is a generalized HIV epidemic (adult prevalence 10.6%) (13) and high tuberculosis (TB) incidence (159 cases per 100,000 population) (14). These factors create a backdrop distinct from most well-resourced settings and common to many countries in the region that is likely to have a substantial bearing on the epidemiology and etiology of CAP; more comprehensive data are therefore crucial to develop appropriate context-specific management guidelines (15). Given the continued rollout of antiretroviral therapy (ART) (16), recent introduction of infant pneumococcal conjugate vaccination (17), and the broader context of rapidly increasing life expectancy and emergence of chronic noncommunicable diseases (18), contemporaneous data are vital. We therefore conducted a prospective study of adults hospitalized with CAP to the largest central hospital in Malawi to describe etiology and to identify risk factors for mortality to inform the development of local guidelines for antibiotic choice and severity assessment. Some of the results of this study have been previously reported in the form of abstracts (19, 20).

## Methods

### Study Setting and Design

We conducted a prospective observational study of adults hospitalized with CAP at Queen Elizabeth Central Hospital, a 1,200-bed teaching hospital that provides free healthcare to the 1.3 million residents of Blantyre district in Southern Malawi. Ethical approval was provided by the Research Ethics Committees of University of Malawi College of Medicine (P.11/12/1309) and Liverpool School of Tropical Medicine (13.02). Some patients participated in linked case-control studies describing the impact of HIV on influenza severity (21) and the association of indoor air pollution exposure with pneumonia (22).

### Study Procedures

We recruited adults (≥18 yr) hospitalized with clinically diagnosed CAP defined as: reported or recorded fever (≥38°C), at least one relevant symptom (cough, chest pain, breathlessness, hemoptysis), and at least one focal chest sign (crepitations, pleural rub, bronchial breathing, percussive dullness, or diminished breath sounds) (adapted from Reference 23). Patients with symptoms for more than 14 days, current antituberculous treatment, or prior admission within the last month were excluded. Further details of the eligibility criteria are provided in the online supplement. Written informed consent was provided by the patient, or in the case of incapacity, by their accompanying guardian.

Patients underwent a standardized clinical assessment on admission and were reviewed daily during hospitalization and then followed at 30 and 90 days after admission to determine vital status. Blood, urine, sputum, and nasopharyngeal aspirate (NPA) were collected at enrolment. Diagnostic pleural aspiration was performed in patients with radiologically confirmed pleural effusion. Clinical care was provided in accordance with local guidelines and directed by the patients’ clinical team.

### Radiographic Assessment

Chest radiographs were performed as early as possible after admission and independently reported by two radiologists and a study clinician using a standardized form. A consensus report of the three assessors was used for analysis. Radiographic pneumonia was defined as the presence of consolidation or other parenchymal abnormality (including reticulonodular change, cavitation, or miliary appearance) or pleural effusion (4). Additional details are provided in the online supplement.

### Laboratory Testing

Hematologic and biochemical analyses were performed by standard methods. HIV status was established by sequential rapid tests (Determine, Alere; and Uni-Gold, Trinity Biotech) (24) and CD4 cell count was performed on FACSCount flow cytometer (Becton Dickinson). Blood cultures were performed using aerobic bottles in the BacT/ALERT 3D automated system (bioMérieux) and isolates identified using standard procedures (25). Urine antigen testing was performed for the detection of *Streptococcus pneumoniae* (BinaxNOW, Alere) and, in a subset of patients, *Legionella pneumophila* (BinaxNOW, Alere). A PCR assay was performed on NPA specimens for the detection of adenovirus; bocavirus; *Chlamydophila pneumoniae*; coronaviruses 229E, HKU1, OC43, and NL63; enterovirus; human metapneumovirus; influenza A and B viruses; *Mycoplasma pneumoniae*; parainfluenza virus types 1–4; parechovirus; respiratory syncytial viruses, and rhinovirus. Xpert MTB/RIF assay (Cepheid) and mycobacterial microscopy and culture (BACTEC MGIT 960 Mycobacterial Detection System, Becton Dickinson) were performed on noninduced sputum specimens. When obtained, pleural fluid samples were sent for Gram stain, aerobic culture, mycobacterial microscopy and culture, and pneumococcal antigen testing. Further details of laboratory methods are provided in the online supplement.

### Statistical Analysis

Statistical analyses were performed with Stata version 12.1 (StataCorp). We tested for differences in continuous variables using Wilcoxon rank sum test, and categorical variables by chi-square test or Fisher exact test as appropriate. Differences in microbial etiology by radiographic status controlling for the effect of HIV were examined using the Mantel-Haenszel method.

Candidate risk factors for 30-day mortality were selected *a priori* based on literature review (26–29). Continuous variables, with the exception of age, were dichotomized at standard cutoff points (29–32). Univariable analyses were performed using logistic regression to explore associations with 30-day mortality. Multivariable models were generated using age, sex, HIV status, and all variables with a *P* value less than 0.2 on univariable analysis, prevalence of at least 5% and for which data were available in more than 95% of patients, and then rationalized by stepwise backward elimination with removal of variables with a *P* value greater than 0.05. The number of covariates in the multivariable model were limited to maintain at least 10 events per variable (33). Subgroup analyses of HIV-infected patients and those with radiographic pneumonia were performed. The association of etiology with mortality was also assessed (*see* online supplement). All available case information was used in each univariable analysis. In multivariable models, we excluded patients with missing data for included variables.

The prognostic performance of several CAP severity-assessment tools, such as CURB65 (29), CRB65 (29), SMRT-CO (simplified version of the SMART-COP tool) (34), modified Infectious Disease Society of America/American Thoracic Society 2007 minor criteria (35), and SWAT-Bp (28), for predicting 30-day mortality was described by calculating sensitivity, specificity, positive and negative predictive values, positive and negative likelihood ratios, and area under the receiver-operating characteristic curve with 95% confidence intervals (CI). Details of how scores were calculated for each severity-assessment tool are provided in the online supplement.

## Results

### Baseline Characteristics

**Figure 1.**
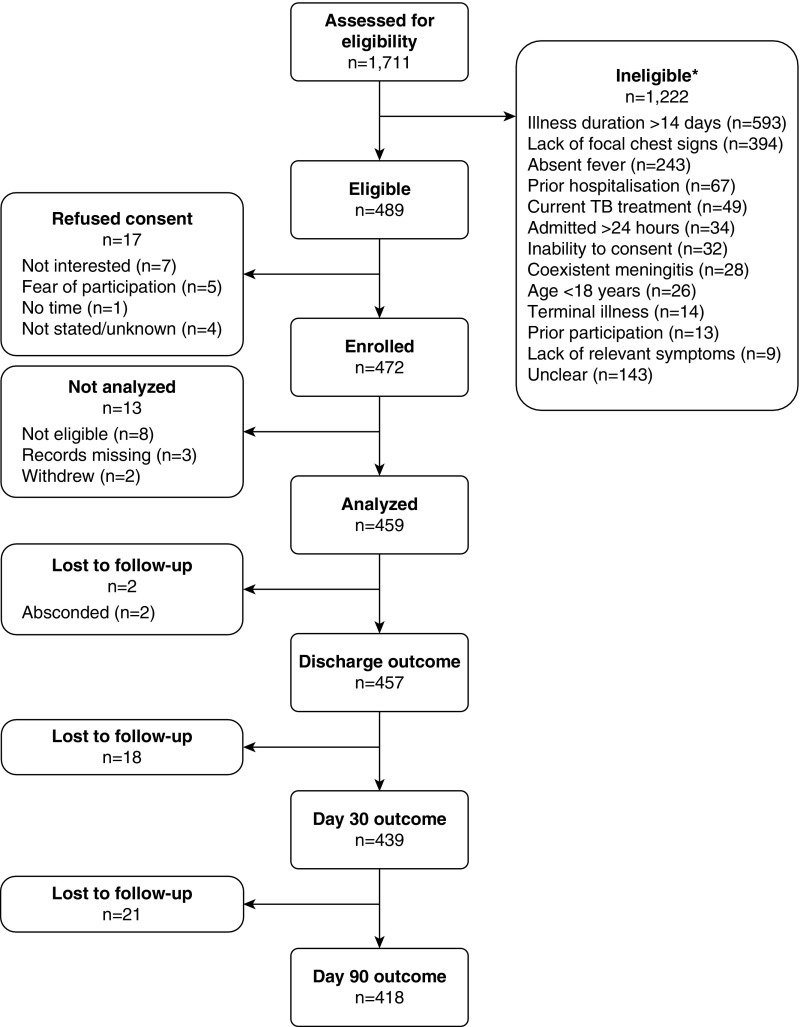
Recruitment and follow-up of a cohort of Malawian adults hospitalized with community-acquired pneumonia. *Some patients were excluded for multiple reasons; hence, the sum of the individual reasons exceeds the total number excluded. TB = tuberculosis.

Between May 15, 2013, and January 31, 2015, we screened 1,711 adult patients, of whom 489 fulfilled the eligibility criteria, 472 were enrolled, and 459 included in the analysis (Figure 1). The median age was 34.7 years (interquartile range, 29.4–41.9) and 285 (62.1%) were male (Table 1; *see* Table E1 in the online supplement). HIV infection was common (355/453; 78.4%; HIV status missing in six individuals) and often newly diagnosed (124/355; 34.9%), whereas other comorbid illnesses were reported infrequently (31/451; 6.9%). A total of 83.3% (189/227) of those known to be HIV-infected reported current ART use. Prior pneumonia was common (108/457; 23.6%) and associated with HIV (odds ratio [OR], 2.91; 95% CI, 1.46–6.30). The median length of admission was 7 days (interquartile range, 4–10).

**Table 1.** Demographic, Clinical, and Radiographic Characteristics of 459 Malawian Adults Hospitalized with Community-acquired Pneumonia

Characteristic	*n* (*%*) of Episodes
Demographics	
Male	285/459 (62.1)
Age*	34.7 (29.4–41.9)
HIV status	
HIV positive^†^	355/453 (78.4)
Newly diagnosed	124/355 (34.9)
CD4 cell count, cells/mm^3^^†^	
All HIV positive*	99 (44–193)
Newly diagnosed HIV positive*	93 (43–179)
ART use in known HIV positive^†^	189/227 (83.3)
Medical history	
Any other comorbid condition	31/451 (6.9)
Chronic lung disease^‡^	15/452 (3.3)
Chronic heart disease^§^	3/452 (0.7)
Hypertension	8/452 (1.8)
Previous tuberculosis^‖^	84/458 (18.3)
Previous pneumonia in last 5 yr^¶^	108/457 (23.6)
Pregnancy	2/174 (1.2)
Current smoker	50/457 (10.9)
Prehospital/clinic attendance and treatment	
Attended another health facility**	283/458 (61.8)
Antibiotics within 2 wk^††^	280/455 (61.5)
Antimalarials within 2 wk	79/457 (17.3)
Traditional remedies within 2 wk	40/456 (8.8)
Baseline observations	
Temperature, °C*	37.9 (37.1–38.9)
<35°C or ≥40°C	14/459 (3.1)
Systolic BP, mm Hg*	106 (93–121)
<90 mm Hg	86/454 (18.9)
Diastolic BP, mm Hg*	68 (59–78)
≤60 mm Hg	143/454 (31.5)
Heart rate, beats/min*	118 (102–132)
≥125 beats/min	176/458 (38.4)
Respiratory rate, breaths/min*	29 (26–34)
≥30 breaths/min	213/446 (47.8)
Oxygen saturations, %*	95 (91–98)
<90 %	73/450 (16.2)
Body mass index, kg/m^2^*	19.9 (18.2–21.8)
<18.5 kg/m^2^	132/444 (29.7)
Baseline laboratory results	
Hemoglobin, g/dl*	11 (9.0–12.8)
<8 g/dl	74/449 (16.5)
White blood cells, ×10^9^ cells/L*	7.7 (5.0–11.4)
<4 × 10^9^ cells/L	67/448 (15.0)
≥15 × 10^9^ cells/L	63/448 (14.1)
Urea, mmol/L*	4.8 (3.3–8.0)
>7 mmol/L	137/450 (30.4)
Creatinine, μmol/L*	76 (59–100)
>120 μmol/L	76/448 (17.0)
Radiologic features^‡‡^	
Radiographic pneumonia	317/417 (76.0)
Consolidation	251/313 (80.2)
Multilobar involvement	73/247 (29.6)
Cavitation	20/317 (6.3)
Pleural effusion	118/315 (37.5)
Outcome^§§^	
Inpatient mortality	51/457 (11.2)
Day 30 mortality	64/439 (14.6)
Day 90 mortality	87/418 (20.8)

*Definition of abbreviations*: ART = antiretroviral therapy; BP = blood pressure.

Data are *n*/*N* (%) unless otherwise indicated. Variation in denominator reflects missing data unless further specified.

* Median and interquartile range are shown.

^†^ HIV status missing in six patients; CD4 count missing in 40 of all with HIV; ART usage missing in four with known HIV.

^‡^ Chronic lung disease includes asthma, chronic obstructive pulmonary disease, and bronchiectasis.

^§^ Chronic heart disease includes congestive cardiac failure, cor pulmonale, and dilated cardiomyopathy.

^||^ Any previous episode of treated tuberculosis regardless of site and confirmation.

^¶^ Any prior episode within the last 5 years of a syndrome compatible with lower respiratory tract infection reviewed in a healthcare facility and treated with antibiotics.

^**^ Included attendance to other hospital, health center, private clinic, traditional healer, or pharmacy.

^††^ Excluded cotrimoxazole prophylaxis in HIV-infected individuals.

^‡‡^ Chest radiographs available in 421 patients; reports based on consensus grading of assessors and denominator may vary when consensus not obtained.

^§§^ Status at hospital discharge, Day 30, and Day 90 missing in 2, 20, and 41, respectively.

Radiographic pneumonia was identified by consensus report in 317 (76.0%) of 417 patients with available interpretable chest radiographs with moderate interobserver agreement (Kappa, 0.75; 95% CI, 0.69–0.81) (Table 1; *see* Table E2). Pleural effusion was identified in 118/416 (28.4%), being the sole basis of defining radiographic pneumonia in 38/315 (12.1%). The proportion of patients with radiographic pneumonia did not vary with HIV status (HIV-infected 247/320 [77.2%] vs*.* HIV-uninfected 66/91 [72.5%]; *P* = 0.36) or with CD4 count (CD4 < 200 cells/mm^3^ 176/222 [79.3%] vs*.* CD4 ≥ 200 cells/mm^3^ 49/64 [76.6%], *P* = 0.64; CD4 count available in 315/355 [88.7%] HIV-infected patients).

### Etiology

Blood for aerobic culture was obtained from 450 of 459 (98.0%) of the participants, NPA from 455/459 (99.1%), and urine specimen for *S. pneumoniae* antigen detection from 433/459 (94.3%) with stored specimens available for *Legionella* antigen testing in 193 (*see* Table E5). At least one sputum specimen for a mycobacterial diagnostic test was obtained from 322/459 (70.2%) participants: smear microscopy, mycobacterial culture, and Xpert MTB/RIF assay were completed in 305/459 (66.4%), 273/459 (59.5%), and 308/459 (67.1%), respectively. A pleural fluid specimen was obtained in 35/459 (7.6%) patients: aerobic bacterial culture, *S. pneumoniae* antigen detection, and mycobacterial smear microscopy and culture were completed in 31, 31, and 35 patients, respectively.

Overall at least one potential pathogen was identified in 278/459 (60.6%) patients including at least one bacterium in 125/452 (27.7%) and at least one virus in 148/459 (32.6%) (Table 2). *S. pneumoniae* was the most commonly identified organism, present in 98 (21.4%) of 458 for whom results of blood culture and/or urinary antigen assay were available, of whom 92 (93.9%) were identified by detection of urinary antigen alone (*see* Table E5). Pneumococcal etiology was more common in HIV-uninfected patients (30/97 [30.9%] vs*.* 68/355 [19.2%]; *P* = 0.01) and those with radiographic pneumonia (83/316 [26.3%] vs. 8/100 [8.0%]; *P* < 0.001) (*see* Table E6), in particular confluent consolidation (OR, 2.51; 95% CI, 1.26–4.98) (*see* Table E7). Overall, 26 (5.8%) of 450 patients for whom blood was sent for culture were bacteremic: *Salmonella enterica* serovar Typhi (*n* = 9), nontyphoidal *Salmonella* (*n* = 7), and *S. pneumoniae* (*n* = 5) were most commonly isolated. *S.* Typhi was identified more frequently in HIV-uninfected patients (5/94 [5%] vs*.* 3/350 [1%]; *P* = 0.02), whereas all cases of nontyphoidal *Salmonella* infection occurred in HIV-infected patients, and both were more common among patients lacking radiographic pneumonia (*see* Table E6). Infection with *M. pneumoniae* (6/455; 1.3%) and *C. pneumoniae* (2/455; 0.4%) were uncommon. None of the 193 patients tested had a positive *Legionella* urinary antigen assay result.

**Table 2.** Etiology of Community-acquired Pneumonia in Malawian Adults Stratified by HIV Status

Organism	All* (*n* = *459*)	HIV Positive (*n* = *355*)	HIV Negative (*n* = *98*)	*P* Value for Difference^†^
*Streptococcus pneumoniae*	98/458 (21.4)	68/355 (19.2)	30/97 (30.9)	0.01
*Salmonella enterica* serovar Typhi	10/450 (2.2)	4/350 (1.1)	5/94 (5.3)	0.02^‡^
Nontyphoidal *Salmonella*^§^	7/450 (1.6)	7/350 (2)	0/94 (0)	0.35^‡^
Other GNEB^‖^	3/450 (0.7)	3/350 (0.9)	0/94 (0)	1.00^‡^
*Staphylococcus aureus*	2/450 (0.4)	1/350 (0.3)	1/94 (1.1)	0.38^‡^
*Mycoplasma pneumoniae*	6/455 (1.3)	6/355 (1.7)	0/98 (0)	0.35^‡^
*Chlamydophila pneumoniae*	2/455 (0.4)	0/355 (0)	2/98 (2.0)	0.05^‡^
*Legionella pneumophila*	0/193 (0)	0/154 (0)	0/38 (0)	—
*Mycobacterium tuberculosis*	75/326 (23.0)	64/257 (24.9)	10/64 (15.6)	0.12
Nontuberculous mycobacteria	8/273 (2.9)	5/217 (2.3)	3/52 (5.8)	0.19^‡^
Influenza viruses	40/454 (8.8)	30/354 (8.5)	9/98 (9.2)	0.83
Adenovirus	35/455 (7.7)	30/355 (8.5)	5/98 (5.1)	0.39^‡^
Coronaviruses	31/455 (6.8)	28/355 (7.9)	3/98 (3.1)	0.11^‡^
Parainfluenza viruses	17/455 (3.7)	15/355 (4.2)	1/98 (1)	0.21^‡^
Rhinovirus	17/455 (3.7)	15/355 (4.2)	2/98 (2)	0.55^‡^
Bocavirus	13/455 (2.9)	13/355 (3.6)	0/98 (0)	0.08^‡^
Metapneumovirus	9/455 (2.0)	8/355 (2.3)	1/98 (1)	0.69^‡^
RSV	8/455 (1.8)	4/355 (1.1)	3/98 (3.1)	0.18^‡^
Enterovirus	5/455 (1.1)	5/355 (1.4)	0/98 (0)	0.59^‡^
Parechovirus	5/455 (1.1)	5/355 (1.4)	0/98 (0)	0.59^‡^
No pathogen detected	181/459 (39.4)	137/355 (38.6)	42/98 (42.9)	0.44

*Definition of abbreviations*: GNEB = gram-negative enteric bacilli; RSV = respiratory syncytial virus.

Data are *n*/*N* (%). Denominators indicate number of patients with at least one relevant test available. *S. pneumoniae* diagnosis based on combination of blood and pleural fluid culture and antigen assay of urine and pleural fluid. *M. tuberculosis* based on combination of sputum microscopy, culture and Xpert MTB/RIF, and pleural fluid culture. Other organisms based on single test. Column totals exceed number of patients because multiple organisms were detected in some.

^*^ Includes six patients with missing HIV status in whom the following organisms were identified: *Salmonella* Typhi, one; *M. tuberculosis*, one; influenza, one; parainfluenza, one; RSV, one; no pathogen detected, two.

^†^ Chi-square test unless otherwise stated.

^‡^ Fisher exact test.

^§^ *Salmonella enterica* serovar Enteritidis and *Salmonella enterica* serovar Typhimurium combined.

^||^ *Escherichia coli* and *Enterobacter cloacae* combined.

Overall, *M. tuberculosis* was identified in 75 (23.0%) of 326 patients who submitted a sputum and/or pleural fluid specimen (Table 2). TB was confirmed by culture in 65/75 (86.7%) and diagnosed on the basis of Xpert MTB/RIF alone, sputum microscopy alone, or pleural fluid microscopy alone in 8/75 (10.7%), 1/75 (1.3%), and 1/75 (1.3%) patients, respectively (*see* Table E5). Nontuberculous mycobacteria were isolated in 8/273 (2.9%) for whom sputum for culture was obtained, of whom one was smear positive. The frequency of TB did not differ significantly between the groups with and without radiographic pneumonia (58/232 [25%] vs*.* 13/68 [19.1%]) (*see* Table E6), although in the latter, TB displaced *S. pneumoniae* as the most commonly identified pathogen, with all cases occurring in HIV-infected patients.

Influenza (40/454; 8.8%), adenovirus (35/455; 7.7%), and coronavirus (31/455; 6.8%) were the most commonly detected viruses. Detection of these respiratory viruses was not associated with HIV status or the presence of radiographic pneumonia (Table 2; *see* Table E6).

**Figure 2.**
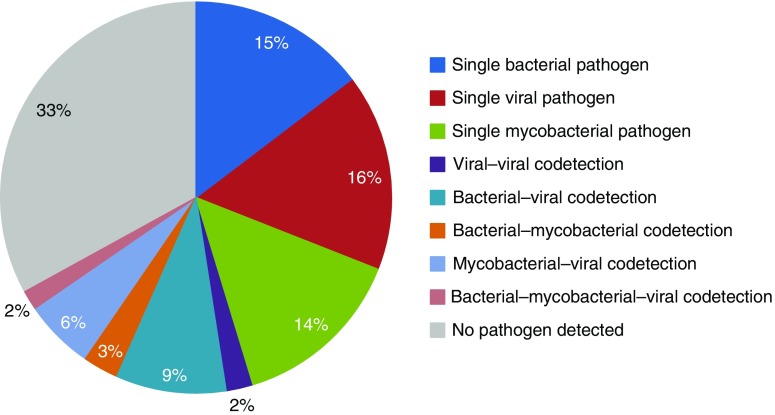
Bacterial, viral, and mycobacterial infection in isolation and combination in Malawian adults hospitalized with community-acquired pneumonia. Etiology defined by blood culture, *Streptococcus pneumoniae* urinary antigen detection, sputum mycobacterial culture and Xpert MTB/RIF, and nasopharyngeal aspirate respiratory pathogen multiplex PCR. Analysis restricted to 307 patients with results available for all tests.

Coinfection relationships were investigated in 307 patients (66.9% of cohort) for whom results for blood culture, pneumococcal urine antigen, sputum mycobacterial culture and/or Xpert MTB/RIF, and NPA PCR were available. At least one organism was detected in 206/307 (67.1%) and coinfection with two or more organisms was present in 67/307 (21.8%) (Figure 2); the combination of bacterial–viral coinfection was most frequent (28/307; 9.1%). *S. pneumoniae* was codetected in one-fifth of patients with influenza (6/31; 19.4%). *M. tuberculosis* was isolated in 9.6% (7/73) of those with *S. pneumoniae* (*see* Table E8). Detection of multiple organisms did not vary with HIV status (54/243 [22.2%] vs*.* 12/62 [19.4%]).

### Mortality

A total of 439/459 (95.6%) patients were successfully followed to 30 days, of whom 64 (14.6%) had died, including 15 and 31 within the first 3 and 7 days following admission, respectively (Table 1). A total of 418 were followed to 90 days, of whom an additional 23 had died. Male sex (adjusted OR [aOR], 2.60; 95% CI, 1.17–5.78), pre-presentation symptom duration greater than 7 days (aOR, 2.78; 95% CI, 1.40–5.54), heart rate greater than or equal to 125 per minute (aOR, 2.99; 95% CI, 1.48–6.06), oxygen saturations less than 90% (aOR, 4.40; 95% CI, 2.03–9.51), and inability to stand (aOR, 3.59; 95% CI, 1.72–7.50) were independently associated with 30-day mortality (Table 3). Neither age nor any underlying comorbid illness, including HIV, or initial antimicrobial treatment was found to be significantly associated with 30-day mortality. Detection of *S. pneumoniae* was associated with reduced mortality (aOR, 0.40; 95% CI, 0.17–0.91) (*see* Table E9), whereas TB was associated with increased mortality (aOR, 2.44; 95% CI, 1.19–5.01). Mortality did not vary with detection of multiple organisms compared with a single organism (5/65 [7.7%] vs. 13/135 [9.6%]; *P* = 0.80).

**Table 3.** Association of Candidate Clinical and Laboratory Risk Factors with 30-Day Mortality in Malawian Adults Hospitalized with Community-acquired Pneumonia

Characteristic	Day 30 Survivors (*n* = *375*)	Day 30 Mortality (*n* = *64*)	Univariable Analysis		Multivariable Analysis	
			OR (95% CI)	*P* Value	aOR (95% CI)	*P* Value
Male	224/375 (59.7)	52/64 (81.3)	2.92 (1.51–5.66)	0.001	2.60 (1.17–5.78)	0.02
Age, yr	**34 (29–42)**	**36 (32–42)**	1.00 (0.98–1.03)	0.73	1.00 (0.97–1.03)	0.93
Current smoker	41/373 (11.0)	7/64 (10.9)	0.99 (0.43–2.33)	0.99	—	—
Regular alcohol use	92/373 (24.7)	23/63 (36.5)	1.76 (1.00–3.09)	0.05	—	—
Unemployed	109/357 (30.5)	13/61 (21.3)	0.62 (0.32–1.18)	0.15	—	—
HIV positive	288/370 (77.8)	54/64 (84.4)	1.53 (0.75–3.15)	0.24	1.26 (0.49–3.21)	0.63
Chronic lung disease	11/371 (3.0)	3/61 (4.9)	1.69 (0.46–6.23)	0.43	—*	—
Chronic heart disease	2/370 (0.5)	1/61 (1.6)	3.07 (0.27–34.35)	0.36	—*	—
Renal disease	0/371 (0)	1/61 (1.6)	—	—	—*	—
Pre-presentation symptoms > 7 d	138/374 (36.9)	36/63 (57.1)	2.28 (1.33–3.92)	0.003	2.78 (1.40–5.54)	0.004
Travel time ≥ 1 h	278/373 (74.5)	51/62 (82.3)	1.58 (0.79–3.16)	0.19	—	—
Previous review	231/375 (61.6)	42/63 (66.7)	1.25 (0.71–2.19)	0.44	—	—
Temperature <35°C or ≥40°C	13/375 (3.5)	1/64 (1.6)	0.44 (0.06–3.44)	0.44	—*	—
Systolic BP < 90 mm Hg	66/371 (17.8)	17/63 (27.0)	1.71 (0.92–3.16)	0.09	—	—
Diastolic BP ≤ 60 mm Hg	112/371 (30.2)	28/63 (44.4)	1.85 (1.07–3.19)	0.03	—^†^	—
Mean arterial BP < 65 mm Hg	46/371 (12.4)	15/63 (23.8)	2.21 (1.14–4.26)	0.02	—^†^	—
Heart rate ≥ 125/min	135/374 (36.1)	37/64 (57.8)	2.43 (1.42–4.16)	0.001	2.99 (1.48–6.06)	0.002
Resp. rate ≥ 30/min	172/364 (47.3)	33/62 (53.2)	1.27 (0.74–2.18)	0.39	—	—
Oxygen saturation < 90%	45/368 (12.2)	27/63 (42.9)	5.38 (2.99–9.70)	<0.0001	4.40 (2.03–9.51)	<0.001
BMI < 18.5 kg/m^2^	108/366 (29.5)	20/61 (32.8)	1.17 (0.65–2.08)	0.61	—^‡^	—
MUAC < 230 mm	103/371 (27.8)	14/63 (22.2)	0.74 (0.39–1.40)	0.36	—	—
Confusion	2/374 (0.5)	3/64 (4.7)	9.15 (1.50–55.87)	0.02	—*	—
Inability to stand	57/375 (15.2)	30/64 (46.9)	4.92 (2.79–8.67)	<0.0001	3.59 (1.72–7.50)	0.001
Hemoglobin< 8 g/dl	55/368 (15.0)	18/61 (29.5)	2.38 (1.28–4.43)	0.006	—	—
White cell count < 4 × 10^9^/L	50/367 (13.6)	15/61 (24.6)	2.07 (1.07–3.98)	0.03	—	—
Platelets < 100 × 10^9^/L	58/368 (15.8)	14/61 (23.0)	1.59 (0.82–3.08)	0.17	—	—
Urea > 7 mmol/L	107/370 (28.9)	27/60 (45.0)	2.01 (1.15–3.51)	0.01	—	—
Creatinine > 120 μmol/L	56/369 (15.2)	15/59 (25.4)	1.91 (0.99–3.65)	0.05	—^§^	—
Glucose ≥ 14 mmol/L	0/330 (0)	0/53 (0)	—	—	—^‖^	—

*Definition of abbreviations*: aOR = adjusted odds ratio; BP = blood pressure; BMI = body mass index; CI = confidence interval; MUAC = mid-upper arm circumference; OR = odds ratio; Resp. = respiratory.

For age (in bold), data are shown as median and interquartile range. Otherwise, data are shown as *n*/*N* (%) with variation in denominator compared with column header reflecting missing data. Univariable and multivariable analyses by logistic regression. For age, OR indicates change in risk of mortality with each year increase. Twenty patients of overall study cohort of 459 excluded because of unknown 30-day outcome. The final multivariable analysis is based on 380 patients with complete results for all included parameters.

^*^ Excluded from multivariable analysis because of prevalence ≤5%.

^†^ Excluded *a priori* from multivariable analysis because of assumed collinearity with systolic blood pressure.

^‡^ Excluded *a priori* because of assumed collinearity with MUAC.

^§^ Excluded *a priori* because of assumed collinearity with urea.

^‖^ Excluded from multivariable analysis because data were missing for >5% of patients.

In a subgroup analysis of the 342 HIV-infected patients followed to Day 30, symptom duration greater than 7 days (aOR, 3.56; 95% CI, 1.60–7.93), heart rate greater than or equal to 125 per minute (aOR, 3.06; 95% CI, 1.40–6.71), oxygen saturations less than 90% (aOR, 2.97; 95% CI, 1.28–6.88), inability to stand (aOR, 4.25; 95% CI, 1.84–9.79), and CD4 count less than 50 cells/mm^3^ (aOR, 2.30; 95% CI, 1.07–4.92) were associated with mortality in multivariable analysis (*see* Table E10). CD4 count less than 200 cells/mm^3^ was not associated with mortality (OR, 1.63; 95% CI, 0.69–3.83). In the subgroup of 305 patients with radiographic pneumonia followed to Day 30, symptom duration greater than 7 days (aOR, 3.30; 95% CI, 1.37–7.96), heart rate greater than or equal to 125 per minute (aOR, 2.66; 95% CI, 1.08–6.57), and multilobar consolidation (aOR, 2.75; 95% CI, 1.17–6.47) were significantly associated with mortality, with a trend toward an association for inability to stand (aOR, 2.63; 95% CI, 1.00–6.89) (*see* Table E11). Thirty-day mortality was, however, significantly higher in the small group of patients for whom a chest radiograph was unavailable (17/36 [47.2%] vs*.* 47/403 [11.7%]; *P* < 0.0001) (*see* Table E3).

### Prognostic Performance of Severity-Assessment Tools

Of the CAP severity-assessment tools examined, SMRT-CO had the highest sensitivity (89.7%; 95% CI, 72.6–97.8), negative predictive value (96.8%; CI, 91.0–99.3%), and overall discriminatory capability (area under receiver-operating characteristic curve, 0.66; 95% CI, 0.57–0.75) for 30-day mortality (Table 4). CURB65 had poor sensitivity and discriminatory capability. Only 38 (8.8%) patients had “severe CAP” by CURB65 (i.e., score ≥ 3) and mortality among those with “mild CAP” (i.e., CURB65 ≤ 1) was 10.8% (29/268). The prognostic performance of each tool was similarly poor in the subgroup with radiographic pneumonia (*see* Table E12).

**Table 4.** Accuracy of Pneumonia Severity-Assessment Tools for Predicting 30-Day Mortality in Malawian Adults Hospitalized with Community-acquired Pneumonia

Score Group	n*	Sensitivity [*% (95% CI)*]	Specificity [*% (95% CI)*]	PPV [*% (95% CI)*]	NPV [*% (95% CI)*]	Positive LR (95% CI)	Negative LR (95% CI)	AUROC (95% CI)
CURB65 ≥ 3	412	14.0 (6.3–25.8)	91.5 (88.2–94.2)	21.1 (9.6–37.3)	86.9 (83.1–90.1)	1.66 (0.80–3.44)	0.94 (0.84–1.05	0.60 (0.52–0.67)
CRB65 ≥ 2	421	27.9 (17.1–40.8)	81.9 (77.6–85.8)	20.7 (12.6–31.1)	87.0 (83.0–90.4)	1.54 (0.97–2.44)	0.88 (0.75–1.04)	0.57 (0.50–0.65)
SMRT-CO ≥ 2	280	89.7 (72.6–97.8)	36.7 (30.7–42.9)	14.1 (9.4–19.9)	96.8 (91.0–99.3)	1.42 (1.21–1.65)	0.28 (0.10–0.83)	0.66 (0.57–0.75)
Modified IDSA/ATS minor criteria ≥ 3	272	48.3 (29.4–67.5)	72.0 (65.9–77.6)	17.1 (9.7–27.0)	92.1 (87.3–95.5)	1.73 (1.13–2.64)	0.72 (0.50–1.03)	0.66 (0.56–0.75)
SWAT-Bp ≥ 3	427	54.1 (40.8–66.9)	68.3 (63.3–73.0)	22.1 (15.8–29.7)	89.9 (85.8–93.2)	1.71 (1.30–2.25)	0.67 (0.51–0.89)	0.65 (0.57–0.72)

*Definition of abbreviations*: AUROC = area under receiver-operating characteristic curve; CI = confidence interval; CRB65 = as per CURB65 with exclusion of urea (29); CURB65 = tool based on presence of confusion, urea > 7 mmol/L, respiratory rate ≥ 30/min, systolic blood pressure < 90 mm Hg and/or diastolic blood pressure ≤ 60 mm Hg, and age ≥ 65 (29); IDSA/ATS = modified version of Infectious Disease Society of America/American Thoracic Society criteria based on presence of respiratory rate ≥ 30/min, oxygen saturations ≤ 90% (used as a surrogate for Pa_O_2__/Fi_O_2__ ratio ≤ 250 criterion included in the published tool), multilobar infiltrates, confusion/disorientation, urea ≥ 7.1 mmol/L, white blood cell count  < 4 × 10^9^ cells/L, platelets < 100 × 10^9^ cells/L, temperature < 36°C, and systolic blood pressure < 90 mm Hg (used as a surrogate for hypotension requiring aggressive fluid resuscitation) (35); LR = likelihood ratio; NPV = negative predictive value; PPV = positive predictive value; SMRT-CO = tool based on presence of systolic blood pressure < 90 mm Hg, multilobar consolidation, respiratory rate ≥ 25/min if ≤50 years or ≥30/min if >50 years, heart rate ≥ 125/min, confusion, and oxygen saturations ≤93% if ≤50 years or ≤90% if >50 years (34); SWAT-Bp = tool based on male sex, wasting, nonambulatory status, temperature <35°C or >38°C, and systolic blood pressure < 100 mm Hg or diastolic blood pressure < 60 mm Hg (28).

For each tool, the performance characteristics displayed are those using the scoring threshold for “severe CAP” as suggested by the authors.

^*^ Score calculated in varying number of patients depending on availability of results for component parameters; SMRT-CO and modified IDSA/ATS minor criteria include a radiologic parameter and thus were only calculated in those with radiographic pneumonia.

## Discussion

In this prospective study of acute CAP in adults that is among the largest conducted in sub-Saharan Africa, we have shown that: the burden of hospitalized pneumonia in adults predominantly remains in young, HIV-infected patients; TB and vaccine-preventable pathogens, such as *S. pneumoniae* and influenza, are common; and mortality is substantial and associated with potentially modifiable risk factors but poorly predicted by widely used CAP severity-assessment tools.

Although completed within the era of ART scale-up, 35% of HIV-infected patients were newly diagnosed, 17% of those with known HIV were not receiving ART, and more than three-quarters had advanced immunosuppression (i.e., CD4 < 200 cells/mm^3^). Further substantial reductions in CAP incidence may therefore be achieved by improved HIV testing and linkage to early initiation of ART (36).

At first glance, the overall crude mortality rate of 14.6% is comparable with that seen in well-resourced settings (26, 29, 37). However, a recent U.K. cohort reported 30-day mortality of 1.5% in patients younger than 50 (38), suggesting a substantially higher age-adjusted mortality rate in our cohort. The identified associations of TB and uncorrected hypoxemia with death, may explain some of this apparent large discrepancy in mortality rate. In keeping with other studies of acute CAP (7, 27, 28), we did not find evidence that HIV overall influenced outcome; increased mortality was evident in only the most profoundly immunocompromised with CD4 cell count less than 50 cells/ml.

Given the observed strong association of hypoxemia with mortality, expansion of supplemental oxygen provision, which across much of sub-Saharan Africa is currently severely limited (39), represents an obvious strategy to be evaluated to improve CAP outcomes. Programmatic interventions in children in low-resource settings based on improved oxygen supply using oxygen concentrators have been associated with a 35% reduction in mortality (40). The lack of association between respiratory rate and mortality underlines the importance of expanding the availability of pulse oximetry in tandem. Similarly, the association of pre-presentation symptom duration with mortality suggests that interventions to improve patient education to alter health care–seeking behavior or referral mechanisms within community healthcare centers should be considered. The continued substantial mortality rate beyond Day 30 also warrants further study to devise targeted interventions to enhance care following discharge from hospital.

The use of objective CAP severity-assessment tools has been shown to improve the recognition of patients with severe CAP likely to require intensive care support (41) and those with mild disease who can be safely managed as outpatients (42). Current South African CAP guidelines recommend that CURB65 is used to inform site of care and initial antimicrobial treatment (43). However, we found that CURB65 showed poor prognostic performance in predicting 30-day mortality in this cohort compared with its performance in CAP cohorts from well-resourced settings (44). This variation likely reflects differences in microbial etiology, demographics, and comorbidity profiles between this cohort and the patient populations in which CURB65 was derived (29), and underlines the importance of prospective validation of prognostic tools in relevant settings (29, 44). The similarly poor performance of the SWAT-Bp score that was derived in a lower respiratory tract infection cohort from Malawi (28), however, highlights the challenges of developing a simple and accurate locally adapted tool. Rather than attempting to derive a single tool capable of accurately predicting mortality across the whole spectrum of disease severity, a tool with high negative predictive performance aimed at identifying those patients with a low risk of adverse outcome suitable for outpatient management may be more feasible and equally useful. In this respect, the SMRT-CO tool performed well with a negative predictive value of 96.8%, but the inclusion of a radiographic parameter (i.e., multilobar involvement) (34) limits its use as a triage tool in settings where chest radiography might not be readily available.

Recent CAP studies corroborate our observation that *S. pneumoniae* remains the commonest identified cause of adult CAP in sub-Saharan Africa (6, 10, 27). Universal infant vaccination with pneumococcal conjugate vaccination was introduced in Malawi in 2011, approximately 2.5 years before the start of recruitment. Following the introduction of pneumococcal conjugate vaccination in the United States and South Africa, rates of adult pneumococcal disease (including pneumonia hospitalizations) fell rapidly as a result of indirect protection caused by reduced pneumococcal transmission from vaccine recipients (45, 46). Further studies are needed to describe the serotype distribution of pneumococcal pneumonia in Malawi, to determine whether targeted vaccination programs of at-risk groups may be beneficial to tackle the persistent burden of adult disease. Recently developed urinary assays that can detect serotype-specific antigens in noninvasive pneumococcal pneumonia may provide a more comprehensive description (47).

When adequate diagnostic tests are performed, TB is identified in a substantial proportion of patients presenting with acute CAP in sub-Saharan Africa with the major burden of disease and associated mortality occurring in HIV-infected individuals (7–9; 27). At 23% of those tested and 16% of the overall cohort, the frequency of TB is similar to that previously reported (7–9; 27), but probably underestimates the true burden given the reliance on spontaneously expectorated sputum specimens and a single specimen submitted for culture. In most sub-Saharan African settings, laboratory diagnosis of TB has until recently relied on sputum smear microscopy that has poor sensitivity, particularly for HIV-associated TB. The World Health Organization has advocated an empirical “step-up” approach, whereby antituberculous treatment is initiated for HIV-infected adults with features of severe respiratory infection and negative sputum smears who fail to improve after 3–5 days of antibacterial treatment (48). Although this approach may limit inappropriate antituberculous treatment, it may miss up to 20% of patients with culture-positive TB and risks delaying treatment in a group of patients with a very high risk of early death (49). The increasingly available Xpert MTB/RIF assay used in this study offers much improved sensitivity and is now recommended as the first-line diagnostic for HIV-associated TB (50). Rapid urine-based diagnostics (e.g., urine Xpert MTB/RIF, mycobacterial glycopeptide lipoarabinomannan assay) may have a complementary role to guide initiation of antituberculous treatment, particularly for acutely ill patients from whom sputum is frequently unobtainable (51, 52).

In keeping with recent CAP studies from well-resourced settings that have used molecular diagnostics (4, 53), we identified high rates of coinfection with multiple organisms, most commonly bacterial–viral coisolation. Defining the clinical significance of each organism detected is challenging, particularly among immunocompromised individuals. Although coisolation may reflect genuine copathogenicity or simply detection of bystander colonizing organisms, more complex relationships in which one organism facilitates the acquisition or pathogenesis of another (e.g., influenza and *S. pneumoniae*) (54) are possible. Bacterial–viral coinfection has been associated with altered levels of innate immune factors (e.g., CRP and IP10) (55) and a more severe clinical course (56). Further work is needed to define the implications for CAP therapy and prevention.

Radiographic facilities are limited in most healthcare facilities in sub-Saharan Africa where the majority of CAP patients are initially managed. Although the clinical case definition used in this study may have missed some patients with radiographic pneumonia who lacked focal signs at the time of recruitment it nonetheless reflects routine clinical practice and usefully provides data to show how the etiologic spectrum of disease varies with chest radiograph findings (57). The association of radiographic changes compatible with infection with *S. pneumoniae* is unsurprising. Although still relatively uncommon, the greater prevalence of invasive *Salmonella* infection among those lacking radiographic pneumonia has important implications for antimicrobial management. Although specific radiologic features were statistically associated with certain pathogens, the marked interobserver variability in interpretation of these features suggests that the clinical utility of this approach is limited.

In recruiting patients exclusively from a single referral hospital, this study shares the limitations common to many CAP observational cohorts. Most patients were seen at another facility before attendance; how they were selected for referral and, correspondingly, the characteristics and outcome of those treated at home is unknown. Hence, the applicability of the findings to community-level settings is potentially limited. Extrapolation of these findings to other countries in sub-Saharan Africa must also be done cautiously because of the potential impact that variations in such factors as TB incidence, HIV prevalence, and climate may have on CAP epidemiology and etiology. Given limitations of the healthcare infrastructure in Malawi, chronic comorbid illnesses may be underdiagnosed. Inconsistencies in drug supply, equipment provision, and staff experience may result in variations in CAP treatment that may impact patient outcome but are not fully accounted for in analyses.

The description of CAP etiology was limited by the lack of sputum microscopy and culture, which may have increased the detection of bacterial pathogens, such as *Haemophilus influenzae* as has been described in previous studies from the region (27, 58). Similarly, we were unable to support invasive respiratory sampling for diagnostic testing for *Pneumocystis jirovecii* and other HIV-associated opportunistic pathogens. The low rate of bacteremia observed may relate to both prehospital antibiotic use and antibiotic initiation before blood couture collection in hospital, which occurred in up to one-third of patients. Interpretation of the significance of respiratory pathogen multiplex PCR results, particularly for respiratory viruses, is hampered by the lack of data from an appropriate control population. The relative contribution of pathogens with seasonal transmission patterns (e.g., influenza) may also have been skewed by a recruitment period that spanned 2 incomplete years.

In conclusion, more than a decade after the introduction of ART, the major burden of CAP in Malawi remains in young patients with HIV rather than the elderly patients with chronic noncommunicable comorbidities that predominate in well-resourced settings. Accordingly, context-specific approaches to severity assessment and defining empirical antimicrobial treatment are needed. The predominance of *S. pneumoniae* and influenza suggests significant opportunities for CAP prevention through targeted vaccination programs. Strategies to encourage prompt patient presentation, to increase early detection and treatment of TB, and to improve supportive care, in particular the correction of hypoxemia, should be evaluated in clinical trials to address the high burden of CAP-related mortality.

## Supplementary Material

Supplements

Author disclosures
