# Supplementary material for: Etiology and Risk Factors for Mortality in an Adult Community-acquired Pneumonia Cohort in Malawi
Source: Am J Respir Crit Care Med. 2019 Aug 1;200(3):359–69. doi: 10.1164/rccm.201807-1333OC (PMC6680311; doi:10.1164/rccm.201807-1333OC)
Supplement: Supplements [file rccm.201807-1333OC.html]

Etiology and Risk Factors for Mortality in an Adult Community-acquired Pneumonia Cohort in Malawi | American Journal of Respiratory and Critical Care Medicine

- aston\_data\_supplement.pdf (1 MB)
- disclosures.pdf (245 KB)
