# Supplementary material for: Etiology and Risk Factors for Mortality in an Adult Community-acquired Pneumonia Cohort in Malawi
Source: Am J Respir Crit Care Med. 2019 Aug 1;200(3):359–69. doi: 10.1164/rccm.201807-1333OC (PMC6680311; doi:10.1164/rccm.201807-1333OC)
Supplement: Supplements [file rccm.201807-1333OC_aston_data_supplement.pdf]

Etiology and Risk Factors for Mortality in an Adult Community-acquired Pneumonia Cohort in Malawi.

Stephen J. Aston, Antonia Ho, Hannah Jary, Jacqueline Huwa, Tamara Mitchell, Sarah Ibitoye, Elizabeth Joekes, Simon Greenwood, Arthur Daire, Jane Mallewa, Dean Everett, Mulinda Nyirenda, Brian Faragher, Henry C. Mwandumba, Robert S. Heyderman and Stephen B. Gordon

Online Data Supplement

SUPPLEMENTARY METHODS

Study design and conduct

We conducted a prospective observational study of adults hospitalised with CAP at Queen Elizabeth Central Hospital (QECH) - a 1200 bed teaching hospital that provides free healthcare to the 1.3 million residents of Blantyre district in Southern Malawi. Recruitment commenced on May 15, 2013 and continued until January 31, 2015. Participant follow-up was completed by May 28, 2015.

Detailed inclusion and exclusion criteria

Clinical aspects of the study were completed by a dedicated team of research nurses and clinical officers who had received extensive training prior to study initiation. Study investigators (SA, AH, HJ, JH and TM) accompanied the clinical study team on their daily rounds at least weekly to evaluate data quality and consistency of data collection. Patients with features of respiratory illness or infection were screened on arrival in the hospital emergency department or on the medical wards within 24 hours of admission. Consenting adults with clinically diagnosed community-acquired pneumonia (CAP) as defined by the eligibility criteria below (summarised in the Methods section) were recruited.

Inclusion criteria

We recruited adults (≥18 years) hospitalised with clinically-diagnosed CAP defined as: reported or recorded fever (≥38°C); at least one relevant symptom (cough, chest pain, breathlessness, haemoptysis); and at least one focal chest sign (crepitations, pleural rub, bronchial breathing, percussive dullness or diminished breath sounds) (E1).

Exclusion criteria

Patients with any of the following were excluded from participation: symptoms for greater than 14 days; suspected co-existent meningitis; pre-admission diagnosis of terminal illness (e.g. metastatic malignancy, terminal AIDS); current anti-tuberculous treatment; admission to hospital more than 24 hours previously; prior hospitalisation within preceding 4 weeks; or prior participation in the study.

Clinical assessment and follow-up

For each participant, the study team completed a standardised clinical assessment consisting of a comprehensive medical history, physical examination and measurement of physiological observations. Information was obtained via direct questioning of the study participant (or accompanying guardian) and by reference to medical notes and health passport. Physiological observations were measured by the study team on admission or abstracted from the medical notes for patients recruited from the medical wards. In lieu of a validated cognitive assessment tool that is not available in Malawi, the presence of confusion was determined using three standardised questions assessing orientation in time, place and person.

Study participants were reviewed on a daily basis each morning until discharge or 14 days post-admission. At the point of hospital discharge or inpatient death, the study team reviewed the clinical notes and recorded details of treatment administered. Patients surviving to discharge were contacted at 30-days and 90-days post admission to determine vital status and hospital readmission. Two attempts were made to obtain this information by phone call to the patient or their guardian; if unsuccessful field workers visited the patient’s home.

Patients with influenza formed the case population of a linked case-control study

describing the impact of HIV on influenza severity (E2). Some patients with radiographic pneumonia who survived to discharge also participated in a further case-control study of the association of exposure to indoor air pollution with the occurrence of pneumonia (E3).

Radiographic assessment

During the period of the study, there was no functional portable radiograph machine available at QECH; consequently, radiographs were not performed in clinically unstable patients that could not be safely transferred to the radiology department. Plain chest radiograph films were photographed on a light-box using a standard light-reflex digital camera mounted on a tripod in a darkened room. All study radiograph reports were generated by review of the set of digital images.

Chest radiographs were reported independently by two study radiologists (EJ and SG) and the study Principal Investigator (SA, an Infectious Diseases Resident Physician). All reporters were blinded to demographic and clinical data at the time of reporting. Radiographs were reported using a standardised form, that was piloted prior to use for study reporting to ensure consistent application. Definitions of radiological features were based on the Fleischner Society: Glossary of terms for thoracic imaging (E4).

Parenchymal abnormalities were categorised as consolidation, reticulonodular change, miliary appearance and cavitation. Consolidation was further characterised in terms of its quality (confluent or patchy), extent (segmental, lobar or multifocal) and distribution (lower, mid- and/or upper zone, or diffuse). Radiographic pneumonia was defined as the presence of consolidation or other parenchymal abnormality (including reticulonodular change, cavitation or miliary appearance) or pleural effusion (E5). Multilobar consolidation was not specifically reported on the form; consolidation was considered to

be multilobar if characterised as diffuse, present in two or more non-contiguous lung zones regardless of extent or present in two or more contiguous lung zones and characterised as multifocal. Multifocal consolidation within a single lung zone was not classified as multilobar. Study analyses were based on a consensus report for each specific feature. If there was disagreement between the reporters, the majority opinion was used. Variability of interpretation was assessed by calculating average agreement and kappa coefficient.

Specimen collection, processing and testing

Haematology and biochemistry

Full blood count was performed on whole blood specimens using a Beckman Coulter HmX Haematology Analyser (Beckman Coulter, California, USA). Whole blood specimens for biochemical assays were centrifuged within 24 hours of collection. Urea and creatinine concentrations were measured in serum specimens using a Beckman Coulter AU480 Chemistry Analyser (Beckman Coulter, California, USA). CD4 cell counts were measured using a Becton Dickinson FACSCount (Becton Dickinson, California, USA) or a Partec CyFlow CD4 Analyser (Sysmex Partec, Görlitz, Germany).

Bacteriology

Blood cultures were performed using standard aerobic bottles in the BacT/ALERT 3D automated system (bioMérieux, Marcy-L'Etoile, France) as previously described (E6). All isolates were identified using standard diagnostic procedures (E7). Coagulase-negative *Staphylococci*, *Bacillus* spp., *Micrococcus* spp. and diphtheroids were considered as contaminants.

Urine was collected in standard containers and refrigerated. The BinaxNOW® urine antigen test for *Streptococcus pneumoniae* (Alere, Massachusetts, USA) was performed in

accordance with the manufacturer's instructions (E8). The same procedure was applied for pleural fluid specimens. The BinaxNOW® test for *Legionella pneumophila* urinary antigen was performed on urine specimens that had been stored at -80°C following collection.

Respiratory pathogen multiplex PCR

The nasopharyngeal aspirate (NPA) specimens were refrigerated immediately following collection and then divided into aliquots before being stored at -80°C in Universal Transport Medium (Copan, Brescia, Italy) for later batch-testing. Total nucleic acids were extracted from 300µl aliquots of each specimen with the Qiagen BioRobotÒ Universal System using the QIAamp One-For-All nucleic acid kit (Qiagen, Manchester, UK). Influenza A and B viruses were detected by real-time reverse transcription polymerase chain reaction (qRT-PCR) using the CDC Human Influenza RT-PCR diagnostic panel (CDC Influenza Division) (E9). Adenovirus, bocavirus, *Chlamydophila pneumoniae*, coronaviruses 229E, HKU1, OC43 and NL63, enterovirus, human metapneumovirus (hMPV), *Mycoplasma pneumoniae*, parainfluenza virus types 1, 2, 3 and 4, parechovirus, respiratory syncytial viruses (RSV) and rhinovirus were detected using the FTD Respiratory Pathogens 33 kit (Fast-track Diagnostics, Luxembourg) (E10). The results for the other organisms detected by the FTD 33 kit were disregarded. 5 or 10 µL of nucleic acid extract was used in each qRT-PCR reaction in combination with the AgPath one-step RT-PCR reagents (Applied Biosystems, Foster City, California, USA). For both kits, PCR conditions were according to the manufacturer's instructions. For the CDC Human Influenza RT-PCR diagnostic panel, samples with cycle threshold (Ct)-value <40 were recorded as positive; for the FTD 33 kit a cycle threshold (Ct)-value <33 was regarded as positive. Appropriate negative and positive control specimens were run alongside each reaction.

Mycobacterial diagnostic tests

Sputum and pleural fluid microscopy were performed according to standard procedures. Briefly, the most viscous part of the sputum specimen was aspirated and expelled onto a dry glass slide, spread to make a thin smear and stained using the Auramine O method. Pleural fluid specimens were concentrated by centrifugation prior to smear preparation. Smear specimens were each examined twice by two independent readers using LED fluorescence microscopy and reported according to standard criteria; discordant results prompted review by a third reader. All sample smears graded as scanty, 1+, 2+, and 3+ were defined as acid-fast bacilli (AFB) smear-positive.

Mycobacterial culture was performed according to standard procedures using the BACTEC MGIT 960 Mycobacterial Detection System (Becton Dickinson Diagnostic Systems, Sparks, Maryland, USA) as previously described (E11, E12). Briefly, following decontamination in sodium hydroxide and concentration by centrifugation, sputum and pleural fluid specimens were inoculated into mycobacterial growth indicator tubes (MGIT) tubes and incubated at 37°C in the BACTEC MGIT automated liquid culture system for up to 44 days. Cultured isolates identified as AFB on ZN microscopy were positively confirmed as *Mycobacterium tuberculosis* complex by microscopic cording and MPT-64 lateral flow assay (Capilia; TAUNS Laboratories, Numazu, Japan). AFB isolates that were negative on either confirmatory test were inoculated onto plain Löwenstein-Jensen (LJ) media and incubated at 25°C, 37°C and 45°C and onto paranitrobenzoic acid (PNB) and incubated at 37°C. Isolates that grew on LJ media at 37°C only were classified as *M. tuberculosis*. Those that grew at 25°C or 45°C or on PNB were classified as nontuberculous mycobacteria and not speciated further. Positive cultures that did not reveal AFB on microscopy were re-cultured using a stored aliquot of the primary

specimen; if the same result was obtained, they were classified as contaminants or false positives.

The Cepheid Xpert MTB/RIF assay (Cepheid, Sunnyvale, California, USA) is a self-contained, fully integrated, automated rapid diagnostic system that uses nested real-time PCR to detect *M. tuberculosis* genomic DNA in sputum and other clinical specimens (E13). The assay was performed in accordance with the manufacturer's instructions (E14).

Assignment of etiology

A bacterial pathogen was determined to be present if: i) detected on blood or pleural fluid culture; ii) *S. pneumoniae* antigen was detected in urine or pleural fluid; iii) *L. pneumophila* antigen was detected in urine; or iv) *C. pneumoniae* or *M. pneumoniae* was detected by PCR in NPA. Certain bacterial blood culture isolates were considered to be contaminants. Mycobacterial infection was determined to be present if detected in sputum or pleural fluid by mycobacterial culture; or if *Mycobacterium tuberculosis* was detected by Xpert MTB/RIF assay. Positive AFB smear microscopy of sputum or pleural fluid in the absence of positive culture or Xpert MTB/RIF assay was assumed to be *M. tuberculosis* infection. Respiratory viral diagnosis was based on detection by PCR in NPA.

Data management

The clinical data were collected in paper-based CRFs and subsequently converted to electronic form using Intelligent Character Recognition scanning software (TeleForm; Cardiff Software Developers, Cardiff, UK). Prior to scanning the CRFs were checked by the clinical team and subsequently by study investigators (SA, AH, HJ and JH) and any discrepancies were queried. Automated validation checks were built in to the character recognition scanning process to identify outliers and erroneous values. Once all data were entered into the study database, further systemic data checks were performed to detect

potential errors, which were checked and corrected to create a final dataset.

Statistical methods

Statistical analyses were performed with Stata version 12.1 (StataCorp; Texas, US). The statistical methods for comparing microbial etiology by radiographic and HIV status and for identifying independent risk factors for 30-day mortality are detailed in the main manuscript. The association of microbial etiology with 30-day mortality for the most commonly identified pathogens (i.e. *S. pneumoniae*, *M. tuberculosis* and influenza) was estimated by multivariable logistic regression analysis with age, sex and HIV status included as covariates.

The inter-observer variability of chest radiograph interpretation between the three readers was assessed by calculating average percentage agreement and kappa coefficient with estimated 95% confidence intervals (E15, E16). The kappa coefficient was interpreted as follow: <0, poor agreement; 0-0.2, slight agreement; 0.21-0.4, fair agreement; 0.41-0.6, moderate agreement; 0.61-0.8, substantial agreement; 0.81-1.0, almost perfect agreement.

CAP severity assessment tools

The following CAP severity-assessment tools were calculated as published and their prognostic performance to predict 30-day mortality was assessed.

- **CURB65:** One point awarded for each of the following components present: confusion; urea >7 mmol/L; respiratory rate ≥30/min; systolic blood pressure <90 mmHg or diastolic blood pressure ≤60 mmHg; age ≥65 years (E17).
- **CRB65:** Derived as per CURB65 with the exclusion of urea >7 mmol/L (E17).
- **SMRT-CO:** Summation of points for each of the following components present: systolic blood pressure <90 mmHg (2 points); multilobar involvement (1 point);

respiratory rate ≥25/min if ≤50 years or ≥30/min if >50 years (1 point); heart rate ≥125/min (1 point); confusion (1 point); oxygen saturations ≤93% if ≤50 years or ≤90% if >50 years (2 points; E18).

- **Modified IDSA/ATS minor criteria:** One point awarded for each of the following components present: respiratory rate ≥30/min; oxygen saturations ≤90% (used as a surrogate for arterial oxygen pressure/fraction of inspired oxygen (PaO_2/FiO_2) ratio ≤250 criterion included in the published tool); multilobar infiltrates; confusion/disorientation; urea ≥7.1 mmol/L; white blood cell count <4 $x10^9$ cells/L; platelets <100 $x10^9$ cells/L; temperature <36°C; systolic blood pressure < 90 mmHg (used as a surrogate for hypotension requiring aggressive fluid resuscitation; E19).
- **SWAT-Bp:** One point awarded for each of the following components present: male sex; wasting; non-ambulatory status; temperature <35°C or >38°C; systolic blood pressure <100 mmHg or diastolic blood pressure <60 mmHg (E20).

To calculate the prognostic performance characteristics of the severity assessment tools, the recommended cutoffs used to define "severe pneumonia" were used: CURB65 ≥3; CRB65 ≥2; SMRT-CO ≥2; modified IDSA/ATS minor criteria ≥3; and SWAT-Bp ≥3. For each severity score, sensitivity, specificity, positive and negative predictive values, positive and negative likelihood ratios and area under the receiver-operating characteristic curve with 95% confidence intervals were calculated using standard methods. Patients with incomplete data were excluded from the analysis.

SUPPLEMENTAL RESULTS

Supplemental Table E1. Demographic, clinical and radiographic characteristics of 459 Malawian adults hospitalised with community-acquired pneumonia.

Characteristic	No. (%) of episodes	
**Demographics**		
Male sex	285/459	(62.1)
Age (yrs)*	34.7	(29.4-41.9)
18-24	53/459	(11.6)
25-34	185/459	40.3)
35-44	136/459	(29.6)
45-54	43/459	(9.4)
56-64	24/459	(5.2)
≥65	18/459	(3.9)
**Socioeconomic factors**		
Employment status		
Paid employment	157/455	(34.5)
Self-employed	152/455	(33.4)
Unemployed	120/455	(26.4)
Other	26/455	(5.7)
Highest educational level attended		
None	45/451	(10.0)
Primary	242/451	(53.7)
Secondary	152/451	(33.7)
Higher	12/451	(2.7)
Main household water source		
River	3/458	(0.7)
Borehole/well	128/458	(28.0)
Public tap/standpipe	247/458	(53.9)
Piped to dwelling	80/458	(17.5)
Difficulty obtaining food		
Often	35/458	(7.6)
Sometimes	202/458	(44.1)
Never	221/458	(48.3)
Main cooking fuel		
Firewood	117/458	(25.6)
Charcoal	298/458	(65.0)
Paraffin	6/458	(1.3)
Electricity	37/458	(8.1)
**HIV status**		
HIV positive†	355/453	(78.4)
Newly diagnosed	124/355	(34.9)
CD4 cell count (cells/mm^3)†		
All HIV-positive*	99	(44-193)
<50	88/315	(27.9)
50-199	158/315	(50.2)
≥200	69/315	(21.9)
Newly diagnosed HIV-positive*	93	(43-179)
ART use in known HIV-positive†	189/227	(83.3)
ART duration†		
<3 months	34/157	(21.7)
3-12 months	35/157	(22.3)
>12 months	89/157	(56.1)

Characteristic	No. (%) of episodes
CPT use on admission[†]	
All HIV-positive	151/355 (42.5)
Known HIV-positive	151/188 (80.3)
**Medical history**	
Any other comorbid condition	31/451 (6.9)
Chronic lung disease[‡]	15/452 (3.3)
Chronic heart disease[§]	3/452 (0.7)
Hypertension	8/452 (1.8)
Cancer	5/452 (1.1)
Chronic kidney disease	1/452 (0.2)
Liver disease	1/452 (0.2)
Stroke	5/452 (1.1)
Epilepsy	1/452 (0.2)
Dementia	2/452 (0.4)
Previous tuberculosis[‖]	84/458 (18.3)
Previous pneumonia in last 5 years[**]	108/457 (23.6)
Pregnancy	2/174 (1.2)
Current smoker	50/457 (10.9)
Regular alcohol intake	122/456 (26.8)
**Pre-hospital attendance & treatment**	
Pre-admission symptom duration[*]	7 (5-12)
1-3 days	63/457 (13.8)
4-7 days	214/457 (46.8)
8-10 days	50/457 (10.9)
11-14 days	130/457 (28.5)
Travel time to hospital	
<1 hour	114/455 (25.0)
1-2 hours	245/455 (53.9)
>2 hours	96/455 (21.1)
Attended another health facility[††]	283/458 (61.8)
Prior review	283/458 (61.8)
Primary health centre	230/283 (81.2)
Private clinic	49/283 (17.3)
Other hospital	7/283 (2.5)
Pharmacy	1/283 (0.4)
Traditional healer	2/283 (0.7)
Antibiotics within 2 weeks[‡‡]	280/455 (61.5)
Antimalarials within 2 weeks	79/457 (17.3)
Traditional remedies within 2 weeks	40/456 (8.8)
**Clinical features**	
Symptoms	
Cough	451/458 (98.5)
Sputum production	357/458 (78.0)
Dyspnoea	440/458 (96.1)
Fever	457/458 (99.8)
Weight loss	277/458 (60.5)
Night sweats	299/457 (65.4)

Characteristic	No. (%) of episodes
Examination features	
Crepitations	387/457 (84.7)
Bronchial breathing	167/458 (36.4)
Pleural effusion	79/457 (17.3)
Confusion/disorientation	5/458 (1.1)
Inability to stand	87/459 (19.0)
Oral thrush	28/458 (6.1)
Kaposi's sarcoma	14/458 (3.1)
**Baseline laboratory results**	
Haemoglobin (g/dL)*	11 (9.0-12.8)
<8	74/449 (16.5)
White blood cells (x 10^9 cells/L)*	7.7 (5.0-11.4)
<4	67/448 (15.0)
≥15	x63/448 (14.1)
Urea (mmol/L)*	4.8 (3.3-8.0)
>7	137/450 (30.4)
Creatinine (µmol/L)*	76 (59-100)
>120	76/448 (17.0)
**Radiological features**[§§]	
Radiographic pneumonia	317/417 (76.0)
Consolidation	251/313 (80.2)
Multilobar involvement	73/247 (29.6)
Cavitation	20/317 (6.3)
Pleural effusion	118/315 (37.5)
**Outcome**[‖‖]	
Inpatient mortality	51/457 (11.2)
Day 30 mortality	64/439 (14.6)
Day 90 mortality	87/418 (20.8)

Definition of abbreviations: ART = antiretroviral therapy; BP = blood pressure; CPT = co-trimoxazole preventative therapy; IQR = interquartile range.
Data are n/N (%) unless indicated by * where median and interquartile range are shown. Variation in denominator reflects missing data unless further specified.
[†]HIV status missing in 6 patients; CD4 count missing in 40 of all with HIV; CPT and ART usage missing in 4 and 43, respectively, with known HIV; ART duration missing in 32 reporting ART use.
[‡]Chronic lung disease includes asthma, COPD and bronchiectasis.
[§]Chronic heart disease includes congestive cardiac failure, cor pulmonale and dilated cardiomyopathy.
[‖]Any previous episode of treated tuberculosis regardless of site and confirmation.
[**]Any prior episode within the last 5 years of a syndrome compatible with lower respiratory tract infection reviewed in a healthcare facility and treated with antibiotics.
[††]Included attendance to other hospital, health centre, private clinic, traditional healer or pharmacy.
[‡‡]Excluded co-trimoxazole prophylaxis in HIV-infected individuals.
[§§]Chest radiographs available in 421 patients; reports based on consensus grading of assessors and denominator may vary when consensus not obtained.
[‖‖]Status at hospital discharge, day 30 and day 90 missing in 2, 20 and 41, respectively.

Supplemental Table E2. Radiological features with inter-report agreement in Malawian adults hospitalised with community-acquired pneumonia.

	Individual scorer's assessment			Consensus interpretation†	All scorer's agreement	
	Radiologist 1 (n=419)*	Radiologist 2 (n=407)*	Clinician (n=417)*		Avg. agree (%)‡	Kappa (95% CI)‡
Radiographic pneumonia	323 (77.1)	297 (73.0)	314 (75.3)	317/417 (76.0)	90.6	0.75 (0.69-0.81)
Any parenchymal abnormality	300 (71.6)	266 (65.3)	268 (64.3)	282/414 (68.1)	86.0	0.68 (0.61-0.74)
Consolidation	245 (58.5)	251 (61.7)	260 (62.4)	251/413 (60.8)	83.3	0.65 (0.59-0.70)
Quality					74.5	0.46 (0.37-0.55)
Confluent	159/244 (65.2)	135/251 (53.8)	161/260 (61.9)	145/233 (62.2)		
Patchy	85/244 (34.9)	116/251 (46.2)	99/260 (38.1)	88/233 (37.8)		
Extent					65.9	0.48 (0.42-0.56)
Segmental	77/245 (31.4)	78/250 (31.2)	83/260 (31.9)	62/225 (27.6)		
Lobar	73/245 (29.8)	73/250 (29.2)	89/260 (34.2)	73/225 (32.4)		
Multifocal	95/245 (38.8)	99/250 (39.6)	88/260 (33.9)	90/225 (40.0)		
Multilobar	85/255 (34.7)	65/251 (25.9)	76/260 (29.2)	73/247 (29.6)	83.3	0.55 (0.46-0.63)
Cavitation	27/417 (6.5)	26/406 (6.4)	15/416 (3.6)	20/418 (4.8)	94.9	0.51 (0.35-0.65)
Reticulonodular change	140 (33.4)	35 (8.6)	24 (5.8)	35/418 (8.4)	76.9	0.15 (0.07-0.24)
Miliary appearance	2/414 (0.5)	2/405 (0.5)	8/416 (1.9)	2/417 (0.5)	98.4	0.17 (-0.01-0.34)
Pleural effusion	111 (26.5)	106 (26.0)	155 (37.2)	118/416 (28.4)	83.7	0.61 (0.55-0.67)
Pneumothorax	2/410 (0.5)	1/406 (0.3)	3/405 (0.7)	2/416 (0.5)	99.3	0.32 (-0.02-0.49)
Mediastinal lymphadenopathy	40 (9.6)	34 (8.4)	50 (12.0)	28/417 (6.7)	87.4	0.30 (0.19-0.42)
Volume loss	76 (18.1)	35 (8.6)	44 (10.6)	30/421 (7.1)	84.1	0.28 (0.17-0.38)
Bronchiectasis	31 (7.4)	8 (2.0)	3 (0.7)	3/421 (0.7)	93.7	0.03 (-0.04-0.11)

Definition of abbreviations: CI = confidence interval.

Data are n/N (%) unless otherwise specified. Chest radiographs were available in 421 of 459 patients; chest radiographs unavailable because of death prior to radiograph (n=14), hospital discharge without radiograph or prior to digital capture (n=18) and unclear reason (n=6).

*Radiologist 1, radiologist 2 and clinician considered 2, 14 and 4 radiographs, respectively, as uninterpretable.

†Consensus values calculated by majority rating of all assessors; denominator may vary when consensus not obtained, most notably for specific features of consolidation which were reported only when assessor regarded consolidation as present.

‡Average agreement and three-way kappa (estimated 95% confidence intervals) calculated for all three assessors.

Supplemental Table E3. Clinical features and outcome by chest radiograph availability and radiographic status in Malawian adults hospitalised with community-acquired pneumonia.

Characteristic	Chest radiograph available (n=417)			Chest radiograph unavailable (n=38)	$P^{‡}$
	Radiographic pneumonia (n=317)	No radiographic pneumonia (n=100)	$P^{†}$		
Male sex	210/317 (66.3)	59/100 (49.0)	0.002	23/38 (60.5)	0.85
Age*	35 (30-42)	33 (27-38)	0.01§	37 (30-44)	0.22§
HIV positive‖	247/313 (78.9)	73/98 (74.5))	0.36	31/38 (81.6)	0.60
Pre-presentation symptoms >7 days	122/316 (38.6)	42/103 (42.0)	0.55	15/37 (40.5)	0.90
Temperature <35 or ≥40 °C	10/317 (3.2)	3/100 (3.0)	1.00**	1/38 (2.6)	1.00**
Systolic BP <90 mmHg	61/314 (19.4)	13/99 (13.1)	0.15	11/37 (29.7)	0.08
Diastolic BP ≤60 mmHg	98/314 (31.2)	26/99 (26.3)	0.35	16/37 (43.2)	0.10
Heart rate ≥125 /min	127/316 (40.2)	35/100 (54.0)	0.35	14/38 (36.8)	0.80
Respiratory rate ≥30 /min	155/307 (50.5)	36/98 (36.7)	0.02	18/37 (48.7)	0.86
Oxygen saturations <90 %	52/308 (16.9)	7/100 (7.0)	0.02	13/38 (34.2)	0.002
BMI <18.5 kg/m^2	94/306 (30.7)	28/98 (28.6)	0.69	10/37 (27.0)	0.69
Inability to stand	51/317 (16.1)	18/100 (18.0)	0.65	16/38 (42.1)	<0.001
Haemoglobin <8 g/dL	45/313 (14.4)	22/96 (22.9)	0.048	6/37 (16.2)	0.98
White blood cells <4 x10^9 /L	41/312 (13.1)	19/96 (19.8)	0.11	7/37 (18.9)	0.49
Urea >7 mmol/L	98/313 (31.3)	27/98 (27.6)	0.48	12/37 (32.4)	0.80
Creatinine >120 µmol/L	46/313 (14.7)	22/96 (22.9)	0.06	7/37 (18.9)	0.72
Day 30 mortality††	31/304 (10.2)	16/98 (16.3)	0.10	17/36 (47.2)	<0.001

Definition of abbreviations: BMI = body mass index; BP = blood pressure
Data are n/N (%) unless indicated by * where median and interquartile range are shown. Variation in denominator compared to column header reflecting missing data.
†Comparison between patients with and without radiographic pneumonia by χ^2 test unless otherwise stated.
‡Comparison between all patients with and all without available interpretable chest radiograph by χ^2 test unless otherwise stated; 4 patients who had chest radiograph but without consensus interpretation excluded from analysis.
§Wilcoxon rank-sum test
‖HIV status missing in 6 patients
**Fisher's exact test
††Status at day 30 missing in 19 patients.

Supplemental Table E4. Intravenous antibiotic treatment duration, length of stay and 30-day mortality by antibiotic treatment group in Malawian adults hospitalised with community-acquired pneumonia.

Antibiotic treatment group	n		Intravenous antibiotic duration, days Median (IQR)		Length of stay, days Median (IQR)		30-day mortality n/N (%)	
Ceftriaxone monotherapy	251/450	(55.8)	5	(3-7)	7	(4-9)	38/242	(15.7)
Ceftriaxone-based combination	114/450	(25.3)	5	(3-7)	8	(5-11)	15/108	(13.9)
Penicillin & Chloramphenicol	65/450	(14.4)	4	(3-6)	6	(4-8)	8/61	(13.1)
Penicillin & Chloramphenicol-based combination	15/450	(3.3)	3	(2-7)	12	(4-18)	0/14	(0)
Amoxicillin (oral)	5/450	(1.1)	-	-	9	(5-11)	1/5	(20)

Definition of abbreviations: IQR = interquartile range.
Patients assigned to treatment group on the basis of all antibiotics prescribed during admission, excluding anti-tuberculous treatment. Patients in ‘combination’ groups received additional intravenous and/or oral agents. Data missing in 8 patients.

Supplemental Table E5. Organism identification by specimen type and diagnostic test stratified by HIV status.

Organism	All[*] (n=459)	HIV-positive (n=355)	HIV-negative (n=98)
**Blood cultures[†]**			
Streptococcus pneumoniae	5/450 (1.1)	4/350 (1.1)	1/94 (1.1)
Salmonella enterica serovar Typhi	9/450 (2.0)	3/350 (0.9)	5/94 (5.3)
Salmonella enterica serovar Typhimurium	6/450 (1.3)	6/350 (1.7)	0/94 (0)
Salmonella enterica serovar Enteritidis	1/450 (0.2)	1/350 (0.3)	0/94 (0)
Staphylococcus aureus	2/450 (0.4)	1/350 (0.3)	1/94 (1.1)
Escherichia coli	2/450 (0.4)	2/350 (0.6)	0/94 (0)
Enterobacter cloacae	1/450 (0.2)	1/350 (0.3)	0/94 (0)
**Pleural fluid culture**			
Salmonella enterica serovar Typhi	1/31 (3.2)	1/19 (5.3)	0/11 (0)
Mycobacterium tuberculosis	7/35 (20.0)	4/22 (18.2)	2/12 (16.7)
***Streptococcus pneumoniae* antigen test**			
Urine	95/433 (21.9)	67/333 (20.1)	28/94 (29.8)
Pleural fluid	3/31 (9.7)	1/20 (5)	2/10 (20)
***Legionella pneumophila* antigen test**			
Urine	0/193 (0)	0/154 (0)	0/38 (0)
**Sputum mycobacterial diagnostic tests**			
AFB smear microscopy	36/305 (11.8)	30/241 (12.4)	6/60 (10.0)
Sputum culture			
Mycobacterium tuberculosis	60/273 (22.0)	54/217 (24.9)	6/52 (11.5)
NTM	8/273 (2.9)	5/217 (2.3)	3/52 (5.8)
Xpert MTB/RIF	56/308 (18.2)	46/244 (18.9)	10/60 (16.7)

Organism	All (n=459)	HIV-positive (n=355)	HIV-negative (n=98)
**Multiplex PCR on nasopharyngeal aspirate**			
Adenovirus	35/455 (7.7)	30/355 (8.5)	5/98 (5.1)
Bocavirus	13/455 (2.9)	13/355 (3.6)	0/98 (0)
Chlamydophila pneumoniae	2/455 (0.4)	0/355 (0)	2/98 (2)
Coronavirus HKU1	3/455 (0.7)	3/355 (0.9)	0/98 (0)
Coronavirus 229E	19/455 (4.2)	16/355 (4.5)	3/98 (3.1)
Coronavirus OC43	6/455 (1.3)	6/355 (1.7)	0/98 (0)
Coronavirus NL63	5/455 (1.4)	5/355 (1.4)	0/98 (0)
Enterovirus	5/455 (1.1)	5/355 (1.4)	0/98 (0)
Influenza A	19/454 (4.2)	13/354 (3.7)	5/98 (5.1)
Influenza B	21/454 (4.6)	17/354 (4.8)	4/98 (4.1)
Metapneumovirus	9/455 (2)	8/355 (2.3)	1/98 (1.0)
Mycoplasma pneumoniae	6/455 (1.3)	6/355 (1.7)	0/98 (0)
Parainfluenza 1	2/455 (0.4)	1/355 (0.3)	1/98 (1.0)
Parainfluenza 2	6/455 (1.3)	6/355 (1.7)	0/98 (0)
Parainfluenza 3	7/455 (1.5)	7/355 (2)	0/98 (0)
Parainfluenza 4	3/455 (0.7)	2/355 (0.6)	0/98 (0)
Parechovirus	5/455 (1.1)	5/355 (1.4)	0/98 (0)
Rhinovirus	17/455 (3.7)	15/355 (4.2)	2/98 (2)
RSV	8/455 (1.8)	4/355 (1.1)	3/98 (3.1)

Definition of abbreviations: NTM = nontuberculous mycobacteria; RSV = respiratory syncytial virus.
Data are n/N (%) specimens positive for specific organism; variation in denominator reflects the availability of the relevant clinical specimen.

[*]In the 6 patients with unknown HIV status the following positive results were obtained: blood culture - *Salmonella* Typhi, 1; pleural fluid culture – *M. tuberculosis*, 1; nasopharyngeal aspirate PCR – 1 each of influenza A, parainfluenza and RSV.

[†]A further 12 blood cultures (3%) yielded organisms regarded as contaminants: coagulase-negative *Staphylococci*, 8; *Micrococcus* spp., 2; *Bacillus* spp., 2.

Supplemental Table E6. Etiology of community-acquired pneumonia in Malawian adults stratified by radiological appearance and HIV status.

Organism	Radiographic pneumonia (n=317)*			No radiographic pneumonia (n=100)*			*P* for difference†
	All	HIV-pos.	HIV-neg.	All	HIV-pos.	HIV-neg.	
Streptococcus pneumoniae	83/316 (26.3)	56/247 (22.7)	27/65 (41.5)	8/100 (8.0)	5/73 (6.9)	3/25 (12)	<0.001
Salmonella enterica serovar Typhi	2/309 (0.7)	2/243 (0.8)	0/62 (0)	8/99 (8.1)	2/72 (2.8)	5/25 (20)	<0.001
Nontyphoidal *Salmonella*‡	1/309 (0.3)	1/243 (0.4)	0/62 (0)	5/99 (5.1)	5/72 (6.9)	0/25 (0)	<0.001
Other GNEB§	2/309 (0.7)	2/243 (0.8)	0/62 (0)	1/99 (1)	1/72 (1.4)	0/25 (0)	0.66
S. aureus	1/309 (0.3)	0/243 (0)	1/62 (1.6)	0/99 (0)	0/72 (0)	0/25 (0)	0.53
Atypical bacteria‖	5/314 (1.6)	4/247 (1.6)	1/66 (1.5)	1/99 (1)	0/73 (0)	1/25 (4)	0.66
Mycobacterium tuberculosis	58/232 (25)	47/186 (25.3)	10/42 (23.8)	13/68 (19.1)	13/50 (26)	0/17 (0)	0.39
NTM	3/194 (1.6)	2/158 (1.3)	1/33 (3)	4/57 (7.0)	2/41 (4.9)	2/15 (13.3)	0.046
Influenza	25/313 (8)	20/246 (8.1)	4/66 (6.1)	11/99 (11.1)	9/73 (12.3)	2/25 (8)	0.26
Adenovirus	22/314 (7)	19/247 (7.7)	3/66 (4.5)	7/99 (7.1)	5/73 (6.9)	2/25 (8)	0.95
Coronavirus	22/314 (7)	19/247 (7.7)	3/66 (4.6)	9/99 (9.1)	9/73 (12.3)	0/25 (0)	0.43
Parainfluenza	10/314 (3.2)	9/247 (3.6)	1/66 (1.5)	6/99 (6.1)	5/73 (6.9)	0/25 (0)	0.34
Rhinovirus	11/314 (3.5)	9/247 (3.6)	2/66 (3)	4/99 (4)	4/73 (5.5)	0/25 (0)	0.76
Bocavirus	9/314 (2.9)	9/247 (3.6)	0/66 (0)	3/99 (3)	3/73 (4.1)	0/25 (0)	0.85
Metapneumovirus	9/314 (2.9)	8/247 (3.2)	1/66 (1.5)	0/99 (0)	0/73 (0)	0/25 (0)	0.09
RSV	4/314 (1.3)	1/247 (0.4)	2/66 (3)	3/99 (3)	2/73 (2.7)	1/25 (4)	0.16
Enterovirus	2/314 (0.6)	2/247 (0.8)	0/66 (0)	1/99 (1)	1/73 (1.4)	0/25 (0)	0.66
Parechovirus	2/314 (0.6)	2/247 (0.8)	0/66 (0)	1/99 (1)	1/73 (1.4)	0/25 (0)	0.66
No pathogen detected	123/317 (38.8)	95/247 (38.5)	26/66 (39.4)	37/100 (37)	25/73 (34.3)	11/25 (48)	0.85

Definition of abbreviations: GNEB = Gram-negative enteric bacilli; NTM = nontuberculous mycobacteria; RSV = respiratory syncytial virus.
Data are n/N (%). Denominators indicate number of patients with at least one relevant test available. *S. pneumoniae* diagnosis based on combination of blood and pleural fluid culture and antigen assay of urine and pleural fluid. *M. tuberculosis* based on combination of sputum microscopy, culture and Xpert MTB/RIF and pleural fluid culture. Other organisms based on single test. *In the 42 patients without available chest radiograph or consensus report for radiographic pneumonia, the following organisms were detected: *S. pneumoniae*, 7; nontyphoidal *Salmonella*, 1; *S. aureus*, 1; atypical bacteria, 2; *M. tuberculosis*, 4; NTM, 1; influenza, 4; adenovirus, 6; parainfluenza, 1; rhinovirus, 2; bocavirus, 1; RSV, 1; enterovirus, 2; parechovirus, 2; no pathogen detected, 21. †Association of organism with radiological appearance controlling for effect of HIV by Mantel-Haenszel χ^2 test. ‡*Salmonella enterica* serovar Enteritidis and *Salmonella enterica* serovar Typhimurium combined. §*Escherichia coli* and *Enterobacter cloacae* combined. ‖*Chlamydophila pneumoniae* and *Mycoplasma pneumoniae* combined.

Supplemental Table E7. Radiological features of community-acquired pneumonia in Malawian adults stratified by microbial etiology.

Radiographic feature*	Organism		
	***Streptococcus pneumoniae*† (n=69)**	***Mycobacterium tuberculosis*‡ (n=51)**	**Influenza (n=16)§**
Any parenchymal abnormality	65/68 (95.6)	43/51 (84.3)	16/16 (100)
Consolidation	61/68 (89.7)	40/51 (78.4)	11/16 (68.8)
Quality§			
Confluent	46/60 (76.7)	17/37 (46.0)	4/9 (44.4)
Patchy	14/60 (23.3)	20/37 (54.1)	5/9 (55.6)
Extent§			
Segmental	11/57 (19.3)	5/37 (13.5)	1/8 (12.5)
Lobar	23/57 (40.4)	8/37 (21.6)	3/8 (37.5)
Multifocal	23/57 (40.4)	24/37 (64.9)	4/8 (50.0)
Multilobar	20/61 (32.8)	20/51 (39.2)	3/14 (21.4)
Cavitation	2/69 (2.9)	6/51 (11.8)	0/16 (0)
Reticulonodular change	4/69 (5.8)	14/51 (27.5)	3/16 (18.8)
Miliary appearance	0/69 (0)	0/50 (0)	0/16 (0)
Pleural effusion	22/69 (31.9)	23/50 (46.0)	3/16 (18.8)
Mediastinal lymphadenopathy	2/69 (2.9)	8/51 (15.7)	1/16 (6.3)

Data area n/N (%). Analysis restricted to patients with radiographic pneumonia who did not have co-infection involving more than one of *S. pneumoniae*, *M. tuberculosis* and influenza to achieve three mutually exclusive groups.

*Based on consensus values of all assessors; denominator may vary when consensus not obtained.

†Additional 6 patients with *M. tuberculosis* and 7 with influenza co-infection excluded. Other co-infections detected in 24: bacterial, 5; viral, 18; mixed viral-non-tuberculous mycobacterial, 1.

‡Additional 6 patients with *S. pneumoniae* and 1 with influenza co-infection excluded. Other co-infections detected in 15: bacterial, 2; viral, 11; mixed viral-bacterial, 2.

§Additional 8 patients with *S. pneumoniae* and 1 with *M. tuberculosis* co-infection excluded. Other co-infections detected in 4: bacterial, 1; viral, 3.

Supplemental Table E8. Matrix showing co-infection combinations in Malawian adults hospitalised with community-acquired pneumonia.

	S. pneumoniae	*S.* Typhi	NTS	Other GNEB	*S. aureus*	Atypical bacteria	*M. tuberculosis*	NTM	Influenza	Adenovirus	Coronaviruses	Parainfluenza	Rhinovirus	Bocavirus	Metapneumovirus	RSV	Enterovirus	Parechovirus	**TOTAL**
S. pneumoniae	40	0	0	0	0	0	7	2	6	7	5	4	6	1	3	2	0	0	**73**
S. Typhi		2	0	0	0	0	1	1	0	0	0	0	0	0	0	0	0	0	**4**
NTS			2	0	0	0	0	2	0	1	1	1	0	0	0	0	0	0	**5**
Other GNEB				0	0	0	0	0	0	0	1	0	0	0	0	0	0	0	**1**
S. aureus					0	0	0	0	1	0	0	0	0	0	0	0	0	0	**1**
Atypical bacteria						1	1	0	0	0	0	1	0	0	0	0	0	0	**3**
M. tuberculosis							42	0	2	8	3	4	3	1	2	1	0	0	**68**
NTM								2	0	2	2	1	1	0	0	0	0	0	**8**
Influenza									19	1	3	1	1	1	0	1	0	0	**31**
Adenovirus										8	1	1	0	0	1	0	0	0	**23**
Coronaviruses											10	0	2	3	0	0	0	0	**25**
Parainfluenza												4	2	0	1	0	0	0	**14**
Rhinovirus													3	0	0	0	1	1	**13**
Bocavirus														3	0	2	0	0	**8**
Metapneumovirus															1	0	0	0	**6**
RSV																2	0	0	**7**
Enterovirus																	0	1	**1**
Parechovirus																		0	**1**

Definition of abbreviations: GNEB = Gram-negative enteric bacilli; NTM = nontuberculous mycobacteria; NTS = nontyphoidal *Salmonella*; RSV = respiratory syncytial virus.
Analysis performed in 307 patients with complete results available for blood culture, pneumococcal urine antigen testing, sputum mycobacterial culture and/or Xpert MTB/RIF, nasopharyngeal aspirate multiplex PCR; 152 excluded for lacking results for one or more of these investigations. Darkly shaded squares indicate mono-infection.

Supplemental Table E9. Association of etiology with 30-day mortality for three commonest pathogens in Malawian adults with community-acquired pneumonia.

Organism	30-day mortality No. (%)	aOR (95% CI)*	*P*
Streptococcus pneumoniae		0.40 (0.17-0.91)	0.03
Yes	7/94 (7.5)		
No	57/344 (16.6)		
Mycobacterium tuberculosis		2.44 (1.19-5.01)	0.02
Yes	17/74 (23.0)		
No	22/241 (9.1)		
Influenza		0.39 (0.09-1.67)	0.20
Yes	2/39 (5.1)		
No	61/396 (15.4)		

Definition of abbreviations: aOR = adjusted odds ratio; CI = confidence interval; IQR = interquartile range.
Data are n/N (%). Status at day 30 determined in 439 of 459 patients overall; individual analyses for each pathogen restricted to those for whom relevant specimens available: *S. pneumoniae* (n=438); *M. tuberculosis* (n=315); influenza (n=435).
*Odds ratios calculated by logistic regression and adjusted for age, sex and HIV status.

Supplemental Table E10. Association of candidate clinical and laboratory risk factors with 30-day mortality in HIV-infected Malawian adults hospitalised with community-acquired pneumonia.

Characteristic	Day 30 survivors (n=288)	Day 30 mortality (n=54)	Univariable analysis OR (95% CI)	Univariable analysis *P*	Multivariable analysis aOR (95% CI)	Multivariable analysis *P*
Male sex	170/288 (59.0)	45/54 (83.3)	3.47 (1.63-7.37)	0.001	1.94 (0.81-4.67)	0.14
Age (yrs)	*34 (30-41)*	*36 (32-41)*	1.01 (0.98-1.03)	0.72	1.00 (0.96-1.04)	0.98
Current smoker	23/286 (8.0)	3/54 (5.6)	0.67 (0.19-2.32)	0.53	-	-
Pre-presentation symptoms >7 days	99/288 (34.4)	32/53 (60.4)	2.91 (1.59-5.31)	0.001	3.56 (1.60-7.93)	0.002
Temperature <35 or ≥40 °C	10/288 (3.5)	1/54 (1.9)	0.52 (0.07-4.18)	0.54	-*	-
Systolic BP <90 mmHg	58/284 (20.4)	15/54 (27.8)	1.50 (0.77-2.90)	0.23	-	-
Heart rate ≥125 /min	117/287 (40.8)	33/54 (61.1)	2.28 (1.26-4.14)	0.007	3.06 (1.40-6.71)	0.005
Resp. rate ≥30 /min)	128/277 (46.2)	28/53 (52.8)	1.30 (0.72-2.35)	0.38	-	-
Oxygen sat$^{\underline{n}}$ <90 %	33/282 (11.7)	23/54 (42.6)	5.60 (2.92-10.73)	<0.0001	2.97 (1.28-6.88)	0.01
MUAC <230 mm	82/285 (28.8)	13/53 (24.5)	0.80 (0.41-1.58)	0.53	-	-
Inability to stand	47/288 (16.3)	26/54 (48.2)	4.76 (2.57-8.84)	<0.0001	4.25 (1.84-9.79)	0.001
Haemoglobin <8 g/dL	51/283 (18.0)	17/51 (33.3)	2.27 (1.18-4.38)	0.01	-	-
White cell count <4 x 10^9/L	41/282 (14.5)	14/51 (27.5)	2.22 (1.11-4.47)	0.03	-	-
Platelets <100 x 10^9/L	48/283 (17.0)	11/51 (21.6)	1.34 (0.64-2.81)	0.43	-	-
Urea >7 mmol/L	90/285 (31.6)	22/50 (44.0)	1.70 (0.92-3.14)	0.09	-	-
CD4 count <50 cells/mm^3	65/257 (25.3)	23/47 (48.9)	2.83 (1.50-5.35)	0.001	2.30 (1.07-4.92)	0.03

Definition of abbreviations: BP = blood pressure; BMI = body mass index; CI = confidence interval; MUAC = mid-upper arm circumference; OR = odds ratio; Resp. = respiratory

For age (in *italics*) data shown as median and interquartile range. Otherwise data shown as n/N (%) with variation in denominator compared to column header reflecting missing data. Univariable and multivariable analyses by logistic regression. For age, odds ratio indicates change in risk of mortality with each year increase. 13 of 355 HIV-infected patients within study cohort excluded due to unknown 30-day outcome. Final multivariable analysis based on 287 patients with complete results for all included parameters.

*Excluded from multivariable analysis because of prevalence ≤5%.

Supplemental Table E11. Association of clinical and laboratory risk factors with 30-day mortality in hospitalised Malawian adults with radiographic community-acquired pneumonia.

Characteristic	Day 30 survivors (n=274)	Day 30 mortality (n=31)	Univariable analysis OR (95% CI)	Univariable analysis *P*	Multivariable analysis aOR (95% CI)	Multivariable analysis *P*
Male sex	179/274 (65.3)	24/31 (77.4)	1.82 (0.76-4.38)	0.18	1.39 (0.53-3.61)	0.51
Age (yrs)	*35 (30-42)*	*37 (32-44)*	1.01 (0.98-1.03)	0.70	1.02 (0.98-1.05)	0.39
Current smoker	28/272 (10.3)	3/31 (9.7)	0.93 (0.27-3.27)	0.92	-	-
Pre-presentation symptoms >7 days	99/273 (36.3)	19/31 (61.3)	2.78 (1.30-5.97)	0.009	3.30 (1.37-7.96)	0.008
HIV-positive	216/271 (79.7)	25/31 (80.7)	1.06 (0.41-2.71)	0.90	1.11 (0.36-3.42)	0.86
Temperature <35 or ≥40 °C	10/274 (3.7)	0/31 (0)	-	-	-[*]	-
Systolic BP <90 mmHg	52/271 (19.2)	7/31 (22.6)	1.23 (0.50-3.00)	0.65	-	-
Heart rate ≥125 /min	104/273 (38.1)	20/31 (64.5)	2.95 (1.36-6.41)	0.006	2.66 (1.08-6.57)	0.03
Resp. rate ≥30 /min)	134/265 (50.6)	16/30 (53.3)	1.12 (0.52-2.38)	0.77	-	-
Oxygen sat^{n} <90 %	42/267 (15.7)	9/30 (30.0)	2.30 (0.98-5.36)	0.06	-	-
MUAC <230 mm	79/270 (29.3)	5/30 (16.7)	0.48 (0.18-1.31)	0.15	-	-
Inability to stand	41/274 (15.0)	10/31 (32.3)	2.71 (1.19-6.17)	0.02	2.63 (1.00-6.89)	0.05
Haemoglobin <8 g/dL	38/270 (14.1)	6/31 (19.4)	1.47 (0.56-3.81)	0.43	-	-
White cell count <4 x 10^9/L	34/269 (12.7)	5/31 (16.1)	1.33 (0.48-3.70)	0.59	-	-
Platelets <100 x 10^9/L	37/270 (13.7)	6/31 (19.4)	1.51 (0.58-3.93)	0.40	-	-
Urea >7 mmol/L	83/270 (30.7)	13/31 (41.9)	1.63 (0.76-3.48)	0.21	-	-
Multilobar	56/266 (21.1)	14/30 (46.7)	3.28 (1.51-7.13)	0.003	2.75 (1.17-6.47)	0.02
Cavitation	18/274 (6.6)	2/31 (6.5)	0.98 (0.22-4.44)	0.98	-	-
Pleural effusion	98/272 (36.4)	16/31 (51.6)	1.86 (0.88-3.93)	0.10	-	-

Definition of abbreviations: BP = blood pressure; BMI = body mass index; CI = confidence interval; MUAC = mid-upper arm circumference; OR = odds ratio; Resp. = respiratory

For age (in *italics*) data shown as median and interquartile range. Otherwise data shown as n/N (%) with variation in denominator compared to column header reflecting missing data. Univariable and multivariable analyses by logistic regression. For age, odds ratio indicates change in risk of mortality with each year increase.

12 of 317 patients with radiographic pneumonia within study cohort excluded due to unknown 30-day outcome. Final multivariable analysis based on 278 patients with complete results for all included parameters.

[*]Excluded from multivariable analysis because of prevalence ≤5%.

Supplemental Table E12. Accuracy of pneumonia severity-assessment tools for predicting 30-day mortality in hospitalised Malawian adults with radiographic community-acquired pneumonia.

Score group	*n*[*]	Sensitivity % (95% CI)	Specificity % (95% CI)	PPV % (95% CI)	NPV % (95% CI)	Positive LR (95% CI)	Negative LR (95% CI)	AUROC (95% CI)
CURB65 ≥3	288	6.7 (0.8-22.1)	89.9 (85.6-93.3)	7.1 (0.9-23.5)	89.2 (84.8-92.7)	0.66 (0.17-2.65)	1.04 (0.94-1.15	0.53 (0.43-0.63)
CRB65 ≥2	292	20.0 (7.7-38.6)	79.8 (74.4-84.5)	10.2 (3.8-20.8)	89.7 (85.1-93.3)	0.99 (0.46-2.10)	1.00 (0.83-1.21)	0.50 (0.39-0.60)
SMRT-CO ≥2	280	89.7 (72.6-97.8)	36.7 (30.7-42.9)	14.1 (9.4-19.9)	96.8 (91.0-99.3)	1.42 (1.21-1.65)	0.28 (0.10-0.84)	0.66 (0.57-0.75)
Modified IDSA/ATS minor criteria ≥3	272	48.3 (29.4-67.5)	72.0 (65.9-77.6)	17.1 (9.7-27.0)	92.1 (87.3-95.5)	1.73 (1.13-2.64)	0.72 (0.50-1.03)	0.66 (0.56-0.75)
SWAT-Bp ≥3	297	40.0 (22.7-59.4)	65.9 (59.9-71.6)	11.7 (6.2-19.5)	90.7 (85.7-94.4)	1.17 (0.73-1.88)	0.91 (0.67-1.23)	0.54 (0.43-0.65)

Definition of abbreviations: AUROC = area under receiver-operating characteristic curve; CI = confidence interval; CURB65 = tool based on presence of confusion, urea >7mmol/L, respiratory rate ≥30/min, systolic blood pressure <90mmHg and/or diastolic blood pressure ≤60mmHg, age ≥65 (E17); CRB65 = as per CURB65 with exclusion of urea (E17); IDSA/ATS = modified version of Infectious Disease Society of America/American Thoracic Society criteria based on presence of respiratory rate ≥30/min, oxygen saturations ≤90% (used as a surrogate for arterial oxygen pressure/fraction of inspired oxygen (PaO_2/FiO_2) ratio ≤250 criterion included in the published tool), multilobar infiltrates, confusion/disorientation, urea ≥7.1 mmol/L, white blood cell count <4 $x10^9$ cells/L, platelets <100 $x10^9$ cells/L, temperature <36°C, systolic blood pressure < 90 mmHg (used as a surrogate for hypotension requiring aggressive fluid resuscitation; E19); LR = likelihood ratio; NPV = negative predictive value; PPV = positive predictive value; SMRT-CO = tool based on presence of systolic blood pressure < 90 mmHg, multilobar consolidation, respiratory rate ≥25/min if ≤50 years or ≥30/min if >50 years, heart rate ≥125/min, confusion and oxygen saturations ≤93% if ≤50 yrs or ≤90% if >50 yrs (E18); SWAT-Bp = tool based on male sex, wasting, non-ambulatory status, temperature <35°C or >38°C and systolic blood pressure <100 mmHg or diastolic blood pressure <60 mmHg (E20). For each tool, the performance characteristics displayed are those using the scoring threshold for "severe CAP" as suggested by the authors. [*]Score calculated in varying number of patients depending on availability of results for component parameters.

REFERENCES

E1. Lim WS, Baudouin SV, George RC, Hill AT, Jamieson C, Le Jeune I, *et al*. BTS guidelines for the management of community-acquired pneumonia in adults: update 2009. *Thorax* 2009; 64 Suppl 3: iii1-55.

E2. Ho A, Aston SJ, Jary H, Mitchell T, Alaerts M, Menyere M, *et al*. Impact of HIV on the burden and severity of influenza illness in Malawian adults: a prospective cohort and parallel case-control study. *Clin Infect Dis* 2017.

E3. Jary HR, Aston S, Ho A, Giorgi E, Kalata N, Nyirenda M, *et al*. Household air pollution, chronic respiratory disease and pneumonia in Malawian adults: A case-control study. *Wellcome Open Res* 2017; 2: 103.

E4. Hansell DM, Bankier AA, MacMahon H, McLoud TC, Muller NL, Remy J. Fleischner Society: glossary of terms for thoracic imaging. *Radiology* 2008; 246: 697-722.

E5. Jain S, Self WH, Wunderink RG, Fakhran S, Balk R, Bramley AM, *et al.*; CDC EPIC Study Team. Community-acquired Pneumonia Requiring Hospitalization among U.S. Adults. *N Engl J Med* 2015; 373: 415-427.

E6. Everett DB, Mukaka M, Denis B, Gordon SB, Carrol ED, van Oosterhout JJ, *et al*. Ten years of surveillance for invasive Streptococcus pneumoniae during the era of antiretroviral scale-up and cotrimoxazole prophylaxis in Malawi. *PLoS One* 2011; 6: e17765.

E7. Barrow GI, Feltham RKA. Cowan and Steel's Manual for the Identification of Medical Bacteria. Cambridge: Cambridge University Press, UK; 1993.

E8. Alere. BinaxNOW Streptococcus pneumoniae Antigen Card Package Insert. Maine, USA: Alere; 2015.

E9. World Health Organization. CDC protocol of realtime RTPCR for influenza A(H1N1). The WHO Collaborating Centre for influenza, CDC Atlanta, US; 2009.

E10. Driscoll AJ, Karron RA, Bhat N, Thumar B, Kodani M, Fields BS, *et al*. Evaluation of fast-track diagnostics and TaqMan array card real-time PCR assays for the detection of respiratory pathogens. *J Microbiol Methods* 2014; 107: 222-226.

E11. Tortoli E, Cichero P, Piersimoni C, Simonetti MT, Gesu G, Nista D. Use of BACTEC MGIT 960 for recovery of mycobacteria from clinical specimens: multicenter study. *J Clin Microbiol* 1999; 37: 3578-3582.

E12. Feasey NA, Banada PP, Howson W, Sloan DJ, Mdolo A, Boehme C, *et al*. Evaluation of Xpert MTB/RIF for detection of tuberculosis from blood samples of HIV-infected adults confirms Mycobacterium tuberculosis bacteremia as an indicator of poor prognosis. *J Clin Microbiol* 2013; 51: 2311-2316.

E13. Lawn SD, Mwaba P, Bates M, Piatek A, Alexander H, Marais BJ, *et al*. Advances in tuberculosis diagnostics: the Xpert MTB/RIF assay and future prospects for a point-of-care test. *Lancet Infect Dis* 2013; 13: 349-361.

E14. Cepheid. Xpert MTB/RIF Package Insert. California, USA: Cepheid; 2015.

E15. Cantor AB. Sample size calculations for Cohen's k. *Psychol Methods* 1996; 1: 150-153.

E16. Reichenheim ME. Sample size for the kappa-statistic of interrater agreement. *Stata Technical Bulletin* 2000; 58: 41-45.

E17. Lim WS, van der Eerden MM, Laing R, Boersma WG, Karalus N, Town GI, *et al*. Defining community acquired pneumonia severity on presentation to hospital: an international derivation and validation study. *Thorax* 2003; 58: 377-382.

E18. Charles PG, Wolfe R, Whitby M, Fine MJ, Fuller AJ, Stirling R, *et al*.; Australian Community-Acquired Pneumonia Study Collaboration. SMART-COP: a tool for predicting the need for intensive respiratory or vasopressor support in community-acquired pneumonia. *Clin Infect Dis* 2008; 47: 375-384.

E19. Mandell LA, Wunderink RG, Anzueto A, Bartlett JG, Campbell GD, Dean NC, *et al*.; Infectious Diseases Society of America; American Thoracic Society. Infectious Diseases Society of America/American Thoracic Society consensus guidelines on the management of community-acquired pneumonia in adults. *Clin Infect Dis* 2007; 44 Suppl 2: S27-72.

E20. Birkhamshaw E, Waitt CJ, Innes M, Waitt PI. Severity assessment of lower respiratory tract infection in Malawi: derivation of a novel index (SWAT-Bp) which outperforms CRB-65. *PLoS One* 2013; 8: e82178.
